# Estrogen, not intrinsic aging, is the major regulator of delayed human wound healing in the elderly

**DOI:** 10.1186/gb-2008-9-5-r80

**Published:** 2008-05-13

**Authors:** Matthew J Hardman, Gillian S Ashcroft

**Affiliations:** 1 Faculty of Life Sciences, University of Manchester, Oxford Road, Manchester M13 9PT, UK

## Abstract

**Background:**

Multiple processes have been implicated in age-related delayed healing, including altered gene expression, intrinsic cellular changes, and changes in extracellular milieu (including hormones). To date, little attempt has been made to assess the relative contribution of each of these processes to a human aging phenomenon. The objective of this study is to determine the contribution of estrogen versus aging in age-associated delayed human wound healing.

**Results:**

Using an Affymetrix microarray-based approach we show that the differences in gene expression between male elderly and young human wounds are almost exclusively estrogen regulated. Expression of 78 probe sets was significantly decreased and 10 probe sets increased in wounds from elderly subjects (with a fold change greater than 7). A total of 83% of down-regulated probe sets and 80% of up-regulated probe sets were estrogen-regulated. Differentially regulated genes were validated at the level of gene and protein expression, with genes identified as estrogen-regulated in human confirmed as estrogen-dependent in young estrogen depleted mice *in vivo*. Moreover, direct estrogen regulation is demonstrated for three array-identified genes, *Sele*, *Lypd3* and *Arg1*, in mouse cells *in vitro*.

**Conclusion:**

These findings have clear implications for our understanding of age-associated cellular changes in the context of wound healing, the latter acting as a paradigm for other age-related repair and maintenance processes, and suggest estrogen has a more profound influence on aging than previously thought.

## Abstract 2

Analysis of gene expression in male elderly and young human wounds suggests that estrogen has a more profound influence on aging than previously thought.

## Background

In elderly subjects wound healing is severely impaired, accompanied by substantial morbidity, mortality and an estimated cost to health services of over $15 billion per annum in the US alone. A unified theory of biological aging is emerging in which cellular maintenance and repair systems are influenced by genes and environment, and wound healing is one of the main pathways of such repair responses [1]. Hormones are potential determining factors in the aging process, and estrogen has been shown to be beneficial in accelerating the age-related impaired tissue repair response in the skin of both genders [2,3]. Elderly male subjects have the highest incidence of chronic non-healing wounds [4,5], correlating with reduced local levels of the beneficial hormone estrogen, with relative maintenance of the androgen hormones that are detrimental to healing [6]. Thus, estrogen has been viewed as a piece of the complex jigsaw modulating aging repair processes. Multiple processes have been implicated in cutaneous aging, including gene expression, intrinsic cellular change and an altered extracellular milieu. However, the relative contribution of each of these processes to age-associated delayed healing is unknown. Here at the level of gene expression, we provide novel insight into the relative contribution of hormones and intrinsic aging, including gerontogenes, to delayed wound healing.

There exists a substantial body of research addressing the tissue, cellular and molecular changes that accompany or directly contribute to aging in a range of model organisms (reviewed in [7]). However, the majority of data, generated in model organisms or *in vitro *(cellular senescence), has yet to be validated in human aging. Moreover the relative contribution of putative gerontogenes to human pathological age-related processes is unknown. Age-associated impaired healing correlates with increased inflammation, increased matrix proteolysis and delayed re-epithelialization leading to chronic wound states, processes modulated by exogenous estrogen treatment [8]. In a recent study we characterized estrogen-regulated changes in gene expression using a model of delayed wound healing in young mice that have been rendered hypogonadal by ovariectomization (hence removing any effects of 'intrinsic aging') [9]. Thus, using comparative analysis we are now in a position to address the relative contributions of estrogen and aging to healing in elderly humans.

Since the major variable contributing to chronic wounds in humans is being an aged male [4,5], our initial approach was to compare acute wound gene expression between young and old male human subjects via Affymetrix microarray. We used the principle of data mining for gene enrichment [10] followed by a cross-species comparison to our recently published dataset of mouse wound estrogen-regulated genes [9] and interrogation of the Dragon online database of estrogen-regulated genes [11] combined with manual annotation to identify estrogen regulated probe sets. Androgen levels, which inhibit healing, are relatively well-maintained in elderly males (data not shown), thus the potential effects are cancelled out when comparing males of different ages. Putative-gerontogenes and genes with established aging-related functions were identified by interrogation of the GenAge online database [12], from aging-associated Gene Ontology (GO) groups and from hand annotation (see Materials and methods/Results for a detailed description of the analysis). We show that the fundamental changes in genes and processes linked to the pathophysiology of age-related delayed healing in humans appear to be almost exclusively estrogen regulated. Estrogen exerts its effects by down-regulating a variety of genes associated with regeneration, matrix production, protease inhibition and epidermal function and up-regulating genes primarily associated with inflammation. These findings have clear implications for our understanding of age-associated cellular changes in the context of wound healing, and are highly relevant with respect to many other age-related repair and maintenance processes.

## Results and discussion

**Figure 1:**
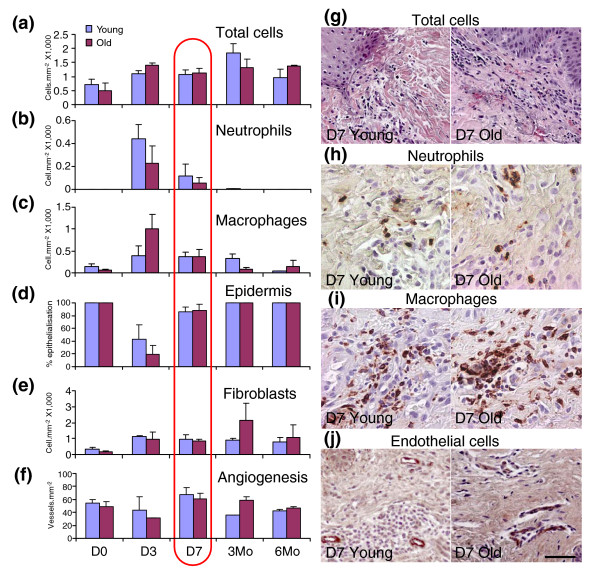
Temporal profile of changes in wound cellular composition. **(a) **Total granulation tissue cell numbers increase over time with no difference between young and old male subjects prior to three months. Closer examination reveals that the inflammatory cell profiles for **(b) **neutrophils and **(c) **macrophages differ significantly at day 3 (D3) post-wounding. **(d) **Differential re-epithelialization is also apparent at this time-point (D3). **(e,f) **In contrast, fibroblast and blood vessel numbers are increased in wounds from elderly subjects at the three month (3Mo) time point. Note equivalent numbers of each cell type in young and old wounds at D7 (red highlight), the time-point chosen for this study. **(g-j) **Comparative images for total cell (hematoxylin and eosin; g), neutrophil (CD15; h) macrophage (CD68; i) and endothelial cell (VWF; j) immunostaining. The scale bar in (j) represents 50 μm (g), 20 μm (h), 35 μm (i), and 45 μm (j).

We initially used immunohistochemical analysis to determine and compare the temporal profile of cellular change in wounds from young and elderly males (Figure 1). We observed clear age-dependent differences in wound numbers of inflammatory cells (neutrophils and macrophages) and rate of re-epithelialization early in healing (three days post-wounding; D3) and fibroblasts/blood vessels during the tissue remodeling phase (three months post-wounding; 3Mo). Crucially, we identified seven days post-wounding (D7) as a period where in males wound cellular composition is equivalent in both young and elderly subjects. This finding facilitated subsequent microarray analysis of wound gene expression by eliminating the possibility of changes in gene expression arising due to disproportionate representation of a specific cell type between biological samples. Hence, changes identified are the result of actual changes in wound gene expression.

**Figure 2:**
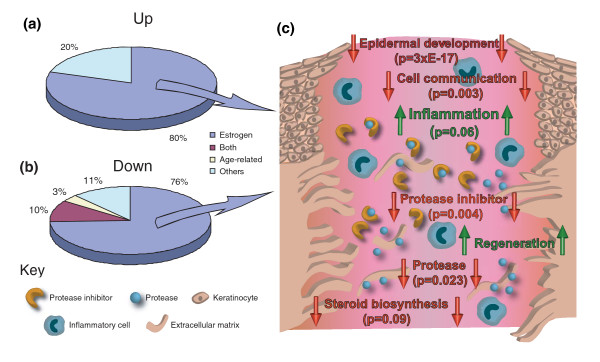
Estrogen-regulated wound-healing-associated genes predominate in age-associated delayed healing. **(a,b)** Graphical representation of the relative proportions of genes significantly up- (a) or down- (b) regulated in wounds from elderly subjects. **(c) **The key overrepresented GO groupings (functionally conserved gene groups) corresponding to each chart segment, their involvement in cutaneous healing, and significance of over-representation (EASE derived *p*-value). The majority of genes in our enriched data set (Additional data file 1) are estrogen regulated and actively involved in cutaneous healing. Ontology groups in red are significantly overrepresented in genes down-regulated in wounds from elderly subjects while those in green are overrepresented in genes with increased expression in wounds from elderly subjects.

For the purpose of this study, probe sets showing significant differential regulation between young and old human wounds were identified by filtering for a fold change of ±7-fold, a q-value <0.1 and expression level >15 (see Additional data file 1 for the full list of identified probe sets; 10 up-regulated and 78 down-regulated). We then used a combination of sources to identify estrogen-regulated genes. We exploited the Dragon online database [11] to assemble a subset of estrogen-regulated genes (subset S1; Additional data file 2). We re-analyzed our own recently published mouse estrogen-regulated gene data set [9] (see Materials and methods) and through comparative analysis identified a gene subset conserved between human and mouse (subset S2; Additional data file 3). A third subset was compiled through hand annotation (subset S3; Additional data file 4). The vast majority of differentially expressed genes were estrogen-regulated (Table 1, Figure 2) and most were down-regulated in wounds from elderly subjects. Using a binomial distribution calculation we determined that our enriched data set contained many more estrogen-regulated genes than would be expected to arise by chance (Dragon: observed = 20, expected = 9.3, *p *= 0.0002; and Mouse data set: observed = 19, expected = 3.8, *p *= 0.0).

**Table 1:** Estrogen-regulated probe sets that are differentially expressed in wounds from elderly compared to young subjects

Affy ID	Gene*	Gene (description)	Function	q-value^†^	FC^‡^
**Down-regulated probe sets (65)**					
207720_at	***LOR***	loricrin	Major cornified envelope protein	0	-235
201909_at	*RPS4Y1*	ribosomal protein S4, Y-linked 1	40S ribosomal component	0	-142
215704_at	*FLG*	filaggrin	Cornified envelope-keratin linker	9.4E-13	-114
206177_s_at	***ARG1***	arginase, liver	Delayed healing-associated	9.2E-11	-82.0
206643_at	*HAL*	histidine ammonia-lyase	Histidine catabolism	1.5E-10	-59.0
206421_s_at	***SERPINB7***	serpin peptidase inhibitor, clade B (ovalbumin), member 7	Proteinase inhibitor for plasmin	5.9E-13	-47.6
206192_at	*CDSN*	Corneodesmosin	Desquamation/adhesion	2.5E-06	-30.1
213796_at	*SPRR1A*	small proline-rich protein 1A	Cornified envelope precursor protein	1.5E-05	-29.4
207324_s_at	***DSC1***	desmocollin 1	Desmosomal cadherin/adhesion	9.6E-06	-28.9
209719_x_at	*SERPINB3*	serpin peptidase inhibitor, clade B (ovalbumin), member 3	Inflammation and cancer-associated	1.6E-05	-22.4
217496_s_at	***IDE***	insulin-degrading enzyme	Wound fluid/resolution of insulin response	4.0E-06	-20.5
211597_s_at	*HOP*	homeodomain-only protein	Serum response factor binding	1.7E-04	-19.7
211726_s_at	*FMO2*	flavin containing monooxygenase 2 (non-functional)	Non-functional oxidative enzyme	7.0E-04	-18.9
220414_at	*CALML5*	calmodulin-like 5	Epidermal-associated calcium-binding	2.0E-05	-17.7
203328_x_at	***IDE***	insulin-degrading enzyme	Wound fluid/resolution of insulin response	1.4E-05	-17.4
210413_x_at	*SERPINB4*	serpin peptidase inhibitor, clade B (ovalbumin), member 4	Cancer and inflammation-associated	3.1E-05	-15.8
219795_at	*SLC6A14*	solute carrier family 6 (amino acid transporter), member 14	Amino acid transport/obesity	6.9E-04	-15.6
210074_at	***CTSL2***	cathepsin L2	Lysosomal cysteine proteinase	3.8E-05	-15.5
222242_s_at	*KLK5*	kallikrein 5	Desquamation, angiogenesis and cancer	4.0E-05	-15.0
201348_at	***GPX3***	glutathione peroxidase 3 (plasma)	Protection from oxidative damage	1.2E-05	-14.8
202018_s_at	*LTF*	lactotransferrin	Inflammatory-cell-derived antioxidant	4.6E-02	-14.5
205185_at	*SPINK5*	serine peptidase inhibitor, Kazal type 5	Anti-inflammatory/anti-microbial	3.8E-05	-14.4
211906_s_at	*SERPINB4*	serpin peptidase inhibitor, clade B (ovalbumin), member 4	Cancer and inflammation-associated	5.7E-05	-12.4
219232_s_at	*EGLN3*	egl nine homolog 3 (*C. elegans*)	Hypoxia-inducible apoptosis-inducing	1.4E-05	-12.1
213256_at	*MARCH3*	membrane-associated ring finger (C3HC4) 3	Poorly characterized ubiquitin ligase	1.6E-05	-12.1
204733_at	***KLK6***	kallikrein 6 (neurosin, zyme)	Hormone regulated serine protease	1.4E-05	-11.9
202179_at	*BLMH*	bleomycin hydrolase	Cysteine peptidase	2.1E-03	-11.8
214549_x_at	*SPRR1A*	small proline-rich protein 1A	Cornified envelope precursor protein	1.6E-04	-11.3
207908_at	*KRT2*	keratin 2 (epidermal ichthyosis bullosa of Siemens)	Supra-basally expressed cytokeratin	1.2E-03	-11.1
210338_s_at	***HSPA8***	heat shock 70 kDa protein 8	ERalpha-inhibiting heat shock protein	9.9E-04	-10.6
209720_s_at	*SERPINB3*	serpin peptidase inhibitor, clade B (ovalbumin), member 3	Inflammation and cancer-associated	3.3E-04	-10.5
201849_at	***BNIP3***	BCL2/adenovirus E1B 19 kDa interacting protein 3	Mitochondrial apoptosis-inducing	2.7E-04	-10.1
205916_at	*S100A7*	S100 calcium binding protein A7	Chemotactic psoriasis-associated protein	1.7E-04	-10.0
204952_at	*LYPD3*	LY6/PLAUR domain containing 3	Upregulated in migrating keratinocytes	1.2E-03	-9.7
206595_at	*CST6*	cystatin E/M	Cysteine protease inhibitor	1.7E-06	-9.3
203327_at	***IDE***	insulin-degrading enzyme	Wound fluid/resolution of insulin response	7.0E-04	-9.3
209555_s_at	***CD36***	CD36 molecule	Thrombospondin receptor	4.0E-03	-9.2
219532_at	*ELOVL4*	elongation of very long chain fatty acids (FEN1/Elo2, SUR4/Elo3, yeast)-like 4	Skin barrier-promoting fatty acid elongase	1.5E-05	-9.2
209126_x_at	*KRT6B*	keratin 6B	Injury-associated keratin	1.7E-03	-9.1
212573_at	*ENDOD1*	endonuclease domain containing 1	Unknown	8.3E-04	-9.0
214599_at	*IVL*	involucrin	Early cornified envelope protein	2.8E-03	-8.8
209218_at	*SQLE*	squalene epoxidase	Rate-limiting sterol biosynthesis enzyme	7.2E-04	-8.8
207356_at	*DEFB4*	defensin, beta 4	Antimicrobial peptide	6.0E-03	-8.8
210138_at	*RGS20*	regulator of G-protein signaling 20	GTPase-activating protein	8.1E-04	-8.7
202504_at	*TRIM29*	tripartite motif-containing 29	Cancer-associated transcription factor	2.2E-03	-8.6
205016_at	*TGFA*	transforming growth factor, alpha	IFN-induced/epidermal regeneration	1.0E-03	-8.5
209309_at	*AZGP1*	alpha-2-glycoprotein 1, zinc	TNFA-regulated prostate-cancer marker	3.5E-04	-8.5
209800_at	*KRT16*	keratin 16 (focal non-epidermolytic palmoplantar keratoderma)	Hyperproliferation and healing-associated keratin	1.2E-03	-8.3
205778_at	*KLK7*	kallikrein 7 (chymotryptic, stratum corneum)	Innate immunity/desquamation	1.2E-05	-8.3
219756_s_at	*POF1B*	premature ovarian failure, 1B	Unknown	3.9E-05	-8.1
214091_s_at	***GPX3***	glutathione peroxidase 3 (plasma)	Protection from oxidative damage	3.0E-03	-8.1
203585_at	*ZNF185*	zinc finger protein 185 (LIM domain)	Actin-associated tumor suppressor	1.4E-03	-8.1
206008_at	*TGM1*	transglutaminase 1	CE formation/epidermal differentiation	4.6E-05	-8.0
202037_s_at	*SFRP1*	secreted frizzled-related protein 1	Repressor of WNT signaling	6.6E-04	-7.9
202539_s_at	*HMGCR*	3-hydroxy-3-methylglutaryl-Coenzyme A reductase	Rate-limiting cholesterol synthesis enzyme	7.4E-04	-7.8
203575_at	*CSNK2A2*	casein kinase 2, alpha prime polypeptide	p53 phosphorylation, WNT signaling	4.6E-04	-7.7
206884_s_at	*SCEL*	sciellin	Cornified envelope precursor protein	2.1E-04	-7.5
204284_at	*PPP1R3C*	protein phosphatase 1, regulatory (inhibitor) subunit 3C	Regulates a wide variety of cellular functions	9.9E-04	-7.4
266_s_at	*CD24*	CD24 molecule	Marker for epithelial neoplasms	2.7E-04	-7.4
203914_x_at	*HPGD*	hydroxyprostaglandin dehydrogenase 15-(NAD)	Main enzyme for prostaglandin degradation	1.6E-04	-7.3
219410_at	*TMEM45A*	transmembrane protein 45A	Hox-regulated/reproductive tissue expressed	8.1E-04	-7.3
206488_s_at	***CD36***	CD36 molecule	Thrombospondin receptor	1.2E-05	-7.3
204881_s_at	*UGCG*	UDP-glucose ceramide glucosyltransferase	Keratinocyte glucosyltransferase	1.8E-03	-7.1
213933_at	*PTGER3*	prostaglandin E receptor 3 (subtype EP3)	Impaired wound healing in null mouse	8.3E-04	-7.1
216379_x_at	*CD24*	CD24 molecule	Marker for epithelial neoplasms	7.9E-04	-7.0
					
**Up-regulated probe sets (8)**					
221728_x_at	***XIST***	X (inactive)-specific transcript	X chromosome inactivation	2.4E-12	191.8
214218_s_at	***XIST***	X (inactive)-specific transcript	X chromosome inactivation	1.0E-09	56.2
206211_at	***SELE***	selectin E (endothelial adhesion molecule 1)	Endothelial-leukocyte adhesion	9.0E-02	8.5
211600_at	***PTPRO***	protein tyrosine phosphatase, receptor type, O	New marker of podocyte injury	5.0E-04	8.4
220940_at	*KIAA1641*	KIAA1641	Unknown	1.0E-04	8.3
203915_at	***CXCL9***	chemokine (C-X-C motif) ligand 9	Interferon induced, TH1 response	6.3E-02	7.3
204324_s_at	*GOLPH4*	golgi phosphoprotein 4	Protein export	8.3E-04	7.3
201205_at	*RRBP1*	ribosome binding protein 1 homolog 180 kDa (dog)	Developmentally regulated extracellular matrix glycoprotein	6.3E-03	7.3

*Genes in bold have been validated by qPCR. ^†^CyberT-derived multiple testing corrected q-value. ^‡^Fold change (old/young).

Down-regulated estrogen-regulated genes were highly enriched for epidermal GO groups, such as epidermal development (EASE *p *= 2.7E-16; Figure 2; Additional data file 5). We observed a strong reduction in epidermal differentiation-associated genes, particularly those encoding cornified envelope proteins (8 genes; EASE *p *= 0.00027), such as *LOR *(235-fold reduction) and *FLG *(114-fold reduction), suggesting a delay in barrier formation. Within hours of injury epithelial cells are mobilized to restore tissue functional integrity. Multiple genes associated with these specific processes are strongly down-regulated in wounds from elderly subjects (Table 1). These include the hyperproliferation-associated keratin, *KRT16 *(8.3-fold reduction) and *LYPD3 *(9.7-fold reduction), a uPAR homologue that is up-regulated in migrating keratinocytes. These findings correlate with the observation that aged keratinocytes show a depressed migratory capacity compared to young cells in a wound environment [13]. Indeed, in wounds from both elderly humans and ovariectomised (ovx) mice re-epithelialization is attenuated (Figure 1) [2,14] and can be restored by topical or systemic estrogen [2,3]. Our data uniquely identify novel gene targets involved in this process.

It has been suggested that delayed wound healing in the elderly results from an imbalance between wound proteases and protease inhibitors, the net result of which is tissue breakdown [8]. Here we demonstrate coordinate changes in expression of estrogen-regulated protease inhibitor encoding genes, including members of the SERPIN family (six probe sets) and cystatin E/M (*CST6*), which act to protect against inappropriate activation of cathepsins. This suggests that delayed-healing wounds are in a profound state of protease inhibitor deprivation (EASE *p *= 0.0038). Novel wound healing genes with dramatic fold differences include *SERPINB7*, which is 47-fold down-regulated in wounds from elderly subjects, and has only previously been reported in the kidney associated with extracellular matrix overexpression [15], and *SERPINB4 *(17-fold down-regulated), the expression of which has, to our knowledge, never been reported in the skin. Skin expression of these novel SERPIN genes is supported by a very high number of skin-derived expressed sequence tags. In this regard, a number of anti-inflammatory, anti-oxidant, and/or anti-microbial genes are also down-regulated in wounds from elderly subjects, such as the antimicrobial peptide defensin beta 4 (*DEFB4*; 8.8-fold), lactoferrin (*LTF*; 14.5-fold), an interesting molecule with antibacterial, antimycotic, antiviral, and anti-inflammatory activity, and secretory leukocyte protease inhibitor (*SLPI*; 5.3-fold), which antagonizes human neutrophil elastase, preventing tissue injury resulting from excessive proteolysis, in addition to possessing broad antimicrobial activity. In *Slpi *null mice increased leukocyte elastase levels lead to severely delayed wound healing with similarities to human chronic wound states [16].

In concordance with the pro-inflammatory aging state, not only is 'inflammatory response' the major GO group overrepresented in the list of genes up-regulated in delayed-healing wounds from elderly subjects (EASE *p *= 0.056), but the endothelially expressed leukocyte adhesion mediator *SELE *displays the second highest fold-change (8.5-fold). *SELE *has previously been shown to be up-regulated in wounds from elderly mice and humans [17]. Moreover, *Sele *null mice display reduced local inflammation [18]. We also observed genes associated with regeneration up-regulated in delayed-healing wounds, including *HOXC6 *(embryonic skin patterning; 5.3-fold) and *TWIST1 *(involved in liver regeneration; 4.5-fold) and in this regard it is intriguing that fetal-like regenerative cutaneous wound repair occurs in the elderly [2]. Insulin degradation in diabetic wounds has been associated with delayed healing [19] and insulin-degrading enzyme (*IDE*) is down-regulated 20-fold in the aged and represented by multiple probe sets, suggesting that increased insulin may have no detrimental effect on wound healing in non-diabetics. Conversely, raised insulin levels have been postulated as a common link in promoting newt limb regeneration [20], which raises the possibility that this pathway is also involved in the reduced scarring phenotype observed in the elderly [2].

Many established wound healing genes are altered in wounds from elderly subjects and are estrogen regulated. Genes with attenuated expression include the classic pro-healing growth factor transforming growth factor alpha (*TGFA*; 8.5-fold down-regulated), genes linked to chronic wound healing, such as arginase 1 (*ARG1*; 82-fold down-regulated), and genes that when knocked out in mice delay healing, such as prostaglandin E receptor 3 (*PTGER3*; 7-fold down-regulated). Such a pronounced reduction in arginase (*ARG1*) expression in wounds from aged subjects is particularly interesting. L-arginine, an essential wound healing amino acid, is converted to nitric oxide, which acts to regulate inflammation. *ARG1 *metabolizes L-arginine to generate proline, a substrate for collagen synthesis. Hence, *ARG1 *is central to modulating the balance between inflammation and matrix deposition, an imbalance in which may explain the dramatic increase in inflammation and decrease in matrix deposition in the aged.

Aging-associated probe sets within our enriched data set were identified by interrogation of a publicly available hand-curated database (the GenAge database) [12] to generate subset S4 (Additional data file 6) or by annotation to known age-associated processes (heat shock, mitochondria, neurodegeneration or response to UV GO groups or by hand annotation) to generate subset S5 (Additional data file 7). Table 2 shows differentially expressed aging-associated genes/probe sets identified in this study, all of which were down-regulated in wounds from elderly subjects. Only a single identified gene, *HSPA8*, is present in the GenAge human aging-related gene list (out of 243 human genes listed in GenAge; Additional data file 6). Moreover, not a single gene orthologue from the model organism GenAge list, which contains 571 genes that have been demonstrated to directly alter life-span in model organisms, is present in our enriched data set.

**Table 2:** Aging-associated probe sets that are differentially expressed in wounds from elderly compared to young subjects

Affy ID	Gene*	Gene (description)	Function	q-value^†^	FC^‡^
**Down-regulated probe sets (12)**					
217496_s_at	***IDE***^§^	insulin-degrading enzyme	Wound fluid/resolution of insulin response	4.0E-06	-20.5
210074_at	***CTSL2***^§^	cathepsin L2	Lysosomal cysteine proteinase	3.8E-05	-15.5
214131_at	***SERPINB13***	serpin peptidase inhibitor, clade B (ovalbumin), member 13	UV-responsive proteinase inhibitor	1.1E-03	-15.0
214131_at	*C12orf5*	chromosome 12 open reading frame 5	Protection from DNA damage	1.1E-03	-12.8
204733_at	***KLK6***^§^	kallikrein 6 (neurosin, zyme)	Hormone regulated serine protease	1.4E-05	-11.9
202179_at	*BLMH*^§^	bleomycin hydrolase	Alzheimer's-associated cysteine peptidase	2.1E-03	-11.8
210338_s_at	***HSPA8***^§^	heat shock 70 kDa protein 8	Aging-associated heat shock protein	9.9E-04	-10.6
201849_at	***BNIP3***^§^	BCL2/adenovirus E1B 19 kDa interacting protein 3	Mitochondrial apoptosis-inducing	2.7E-04	-10.1
203328_x_at	***IDE***^§^	insulin-degrading enzyme	Wound fluid/resolution of insulin response	1.4E-05	-17.4
203327_at	***IDE***^§^	insulin-degrading enzyme	Wound fluid/resolution of insulin response	7.0E-04	-9.3
205016_at	*TGFA*^§^	transforming growth factor, alpha	IFN-induced/epidermal regeneration	1.0E-03	-8.5
212907_at	***SLC30A1***	Solute carrier family 30 (zinc transporter), member 1	Zinc/calcium ion transporter	8.5E-04	-7.3

*Genes in bold have been validated by qPCR. ^†^CyberT-derived multiple testing corrected q-value. ^‡^Fold change (old/young). ^§^Also estrogen-regulated (Table 1).

In light of the considerable overrepresentation of estrogen-regulated genes identified in this study, we next asked whether there was statistically significant enrichment for age-associated genes. Using a binomial distribution we calculate that, based on the size of the human GenAge database (243 genes), the total number of genes on the U133 array (13,290) and the total number of genes in our data set (78), we would expect our enriched data set to contain 1.4 genes from the GenAge database purely by chance. Hence we observe a surprising, non-statistically significant (*p *= 0.72) under-representation of aging-associated genes. For this binomial calculation we have deliberately excluded the much larger list of GenAge genes shown to modulate lifespan in animal models, because of obvious orthologue issues. Including the full GenAge list gave a figure of 3.6 genes expected by chance (*p *= 0.16). Notably, *HSPA8*, the gene that we identified as being present in the GenAge database, is also estrogen-regulated. Indeed, 76% of the aging-related genes identified in this study were additionally estrogen-regulated. Hence, it follows that the most likely candidate genes for mediating intrinsic aging-associated effects on healing are directly estrogen-regulated. This observation underpins the key finding of this study, namely that estrogen-mediated changes in gene expression are central to age-associated delayed healing.

In an attempt to specifically identify further animal-model derived putative-human gerontogenes, we relaxed our array filtering criteria. Filtering for fold change (±1.5-fold), *p*-value (<0.05) and expression level (>15) identified 20 genes from either the human or model organisms GenAge database (Additional data file 8). Again, this constituted under-representation, which in this instance was highly significant (*p *= 0). Most noticeably we found that every identified putative-life span modulating gene (i.e., gerontogene) up-regulated in elderly human wounds acts to extend life-span in animal models (Additional data file 8). The observed beneficial effects of these genes in animal models are at odds with the detrimental nature of delayed human healing, again reinforcing the lack of importance of gerontogenes in the process. In contrast, while some down-regulated putative lifespan modulating genes (i.e., gerontogenes) were associated with extended lifespan (9 out of 14) others were associated with reduced lifespan (5 out of 14).

Those genes not regulated by estrogen nor classed as aging-associated (Table 3) were involved in diverse functions, such as energy supply and protein catabolism (20% of up-regulated and 11% of down-regulated genes; Figure 2) or were of unknown function (36% of genes) and could not, therefore, have been assigned to estrogen or age-associated gene lists.

**Table 3:** Non-aging and non-estrogen-associated probe sets that are differentially expressed in wounds from elderly compared to young subjects

Affy ID	Gene	Gene (description)	Function	q-value*	FC^†^
**Downregulated probe sets (10)**					
205000_at	*DDX3Y*	DEAD (Asp-Glu-Ala-Asp) box polypeptide 3, Y-linked	Male fertility-associated RNA helicase	5.9E-13	-78.6
217521_at	*N54942*	Transcribed locus	Unknown	1.1E-05	-20.1
213780_at	*TCHH*	Trichohyalin	Hair follicle/cornified envelope	1.0E-02	-13.8
220322_at	*IL1F9*	interleukin 1 family, member 9	Keratinocyte cytokine	9.7E-04	-9.9
218454_at	*FLJ22662*	hypothetical protein FLJ22662	Unknown	1.2E-03	-9.9
218150_at	*ARL5A*	ADP-ribosylation factor-like 5A	Developmentally regulated nuclear protein	1.8E-03	-9.0
205001_s_at	*DDX3Y*	DEAD (Asp-Glu-Ala-Asp) box polypeptide 3, Y-linked	Male fertility-associated RNA helicase	1.1E-05	-8.6
214131_at	*CYorf15B*	chromosome Y open reading frame 15B	X-degenerate gene	1.1E-03	-8.1
203180_at	*ALDH1A3*	Aldehyde dehydrogenase 1 family, member A3	Detoxification of aldehydes	7.8E-03	-8.0
207602_at	*TMPRSS11D*	transmembrane protease, serine 11D	Psoriasis-associated serine protease	2.3E-04	-7.9
					
**Upregulated probe sets (2)**					
213369_at	*PCDH21*	protocadherin 21	Adhesion	1.3E-05	11.9
221501_x_at	*LOC339047*	hypothetical protein LOC339047	Unknown	9.8E-05	9.3

*CyberT-derived multiple testing corrected q-value. ^†^Fold change (old/young).

**Figure 3:**
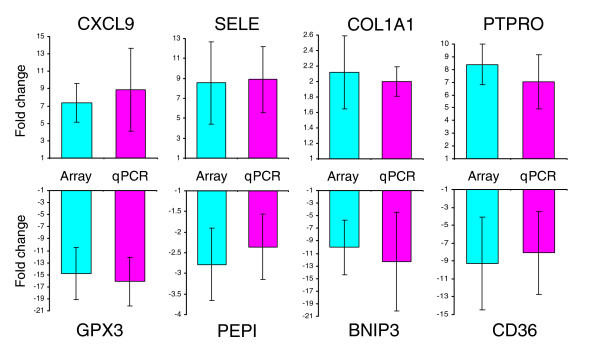
Validation of array-determined gene expression change by qPCR. Data are represented as fold change (old/young) for array data (blue) and qPCR data (pink). Results are presented as mean ± standard error of the mean; n = 3 for arrays and n = 5 for qPCR.

**Figure 4:**
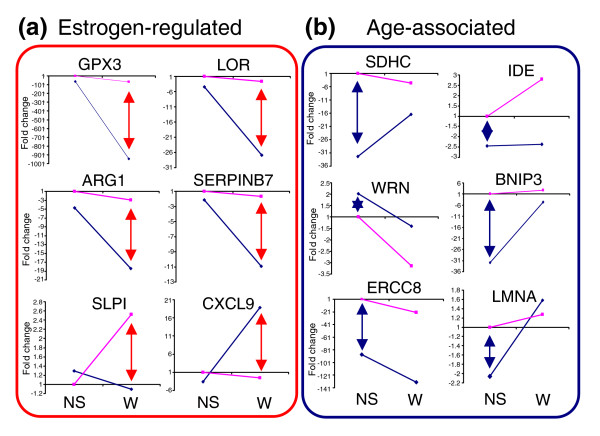
Quantification of gene expression change by qPCR. Comparison of gene expression between normal skin (NS) and wounds (W) from young (pink) and old (blue) human subjects. **(a) **All estrogen-regulated genes tested displayed clear differential wound expression (red double-ended arrow) with more parity of expression in normal skin. **(b) **In contrast, genes identified as age-associated displayed pronounced changes in normal skin (blue double-ended arrow) of similar or greater magnitude than the wound gene expression change. n = 3-5 per group.

**Figure 5:**
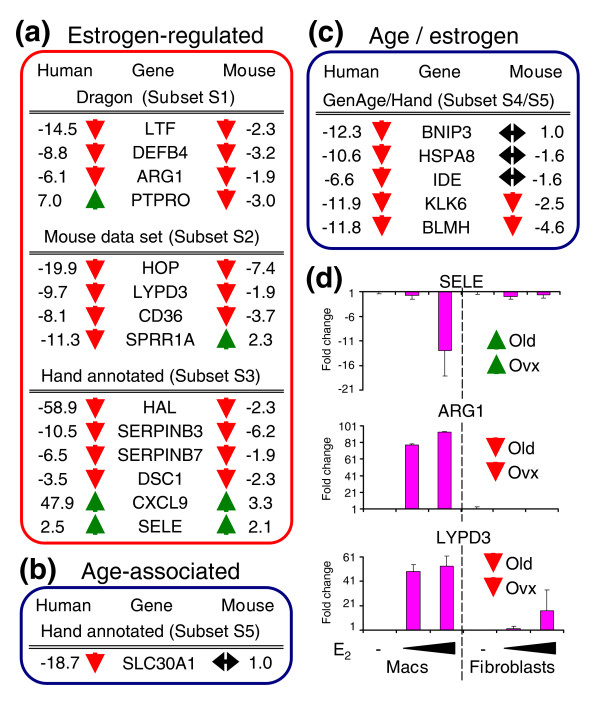
Gene expression changes identified in aged males are mirrored in ovx female mice. **(a,b) **Comparison of wound gene expression change between human males (old/young; left) and estrogen-deprived young female mice (ovx/intact; right). Arrows indicate direction of change, green up and red down. (a) Estrogen-regulated genes are similarly changed in both mouse and human. (b) In contrast, a gene identified as age-associated is unchanged in wounds from ovx female mice. **(c) **Genes categorized as both estrogen-regulated and age-associated were either unchanged in mouse (indicating predominant age-association) or similarly changed in both mouse and human (indicating predominant estrogen-regulation). **(d) **Demonstration that selected array-identified genes are directly estrogen regulated in mouse primary macrophages and/or fibroblasts *in vitro*. Results are presented as mean ± standard error of the mean; n = 3-6 per group.

In order to validate our data, primers were designed to 27 of the key genes identified in this study and quantitative real-time PCR (qPCR) carried out on the same wound samples as used for the arrays and on additional wound samples. In all cases the real-time findings confirmed the array results (Figure 3 and data not shown). We then examined the expression of these genes by qPCR in normal skin and wounds to determine whether the observed changes were present prior to wounding or were specifically induced by wounding (Figure 4 and data not shown). Genes fell into two distinct groups segregating depending on estrogen-regulation or age-association. All estrogen-regulated genes displayed a statistically significant difference in expression between wounds from young and old subjects with a far lower magnitude difference in normal skin (Figure 4a; for example, *LOR*), indicating that the major effects of estrogen are on injured tissue. In contrast, all age-associated genes displayed pronounced change between old and young normal skin in addition to, and often of greater magnitude than, the wound (Figure 4b; for example, *SDHC*), suggesting that age-associated change precedes the healing response. Whilst this does not preclude such genes from influencing subsequent healing responses, our data suggest that not only does estrogen regulate the vast majority of genes involved in healing, but that the gene profiles mimic those seen in wounds from estrogen-deprived young animals (Figure 5a). Of 14 estrogen-regulated genes (selected from human subsets S1, S2 and S3), 12 (86%) were significantly changed in the same direction between human and mouse (Figure 5a). The remaining genes (*PTPRO *and *SPRR1A*) were also significantly changed in both human and mouse but in opposite directions. We next tested selected genes for direct estrogen regulation *in vitro *(Figure 5d and data not shown). *SELE*, which is increased in both old human and ovx mouse wounds, was down-regulated by estrogen *in vitro*, while *LYPD3 *and *ARG1*, decreased in both old human and ovx mouse wounds, was up-regulated by estrogen. Changes in gene expression were seen predominantly in macrophages reinforcing the role of inflammation in age-associated delayed healing.

Moreover, we reasoned that as both mouse groups (intact and ovx) were of equal age (ten weeks) then genes identified in human as age-associated should be unchanged upon mouse comparison. This was confirmed for *SLC30A1*, a gene identified as age-associated but not estrogen-regulated in human (Figure 5b; 1.0-fold), and an additional three genes identified in human as both age-associated and estrogen-regulated (Figure 5c; *BNIP3*, *HSPA8 *and *IDE*). Wound expression of all three genes was not significantly altered between ovx and intact mice, indicating predominant association with age as opposed to estrogen status.

**Figure 6:**
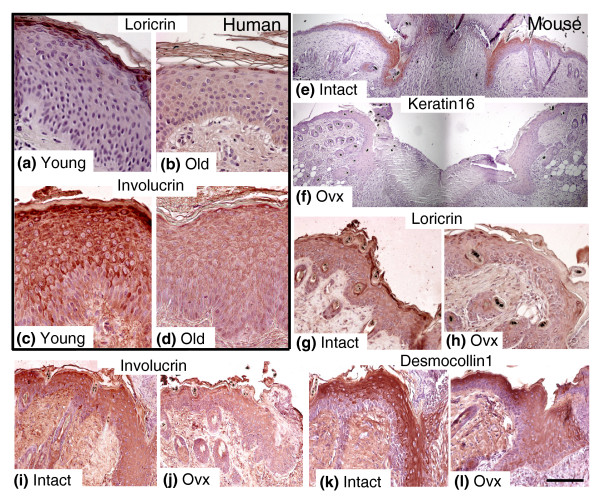
Immunohistochemical analysis of epidermal proteins encoded by array-identified estrogen-regulated genes demonstrates altered expression in wounds from both old humans and ovx young female mice. **(a-d) **Representative immunohistochemical localization of the epidermal differentiation markers loricrin and involucrin demonstrates decreased expression in wound epidermis from old males compared to wounds from young males. **(e-l) **Representative immunohistochemical localization of the epidermal proteins keratin 16, loricrin, involucrin and desmocollin 1, which are decreased in wound epidermis from young ovx mice compared to wounds from mice with intact ovaries. The scale bar in (l) represents 70 μm (a-d), 300 μm (e-f), and 140 μm (g-j).

We next asked whether observed changes in gene expression translated into equivalent changes in wound protein levels. As epidermal genes were most significantly overrepresented in our enriched human data set we initially focussed on expression of key epidermal proteins (Figure 6). We selected the terminal differentiation markers loricrin (-235-fold gene expression) and involucrin (-8.8-fold gene expression), the desmosomal cadherin democollin1 (-28.9-fold gene expression) and the injury-associated intermediate filament protein keratin16 (-8.3-fold gene expression). In agreement with gene expression change, both loricrin and involucrin protein levels were reduced in suprabasal wound epidermis from elderly human subjects (Figure 6a-d). In addition, the estrogen-regulation was confirmed at the protein level by reduced expression of all four proteins in wound epidermis from ovx female mice compared to intact mice (Figure 6e-l). The difference in keratin 16 expression between intact and ovx mouse wounds was particularly striking (compare Figure 6e to 6f). We annotated keratin 16 as estrogen regulated (subset S3; Additional data file 4) based on its inclusion on the EstrArray custom estrogen-regulated gene microarray [21]. To our knowledge, this study provides the first demonstration of keratin 16 (*KRT16*) regulation by estrogen *in vivo*. Moreover, a pronounced lack of KRT16 in the wound edge epidermis from ovx mice is entirely novel and may represent an important contributing factor to delayed re-epithelialization, as keratin 16-mediated re-organization of intermediate filaments in wound edge keratinocytes has been proposed to facilitate re-epithelialization [22].

**Figure 7:**
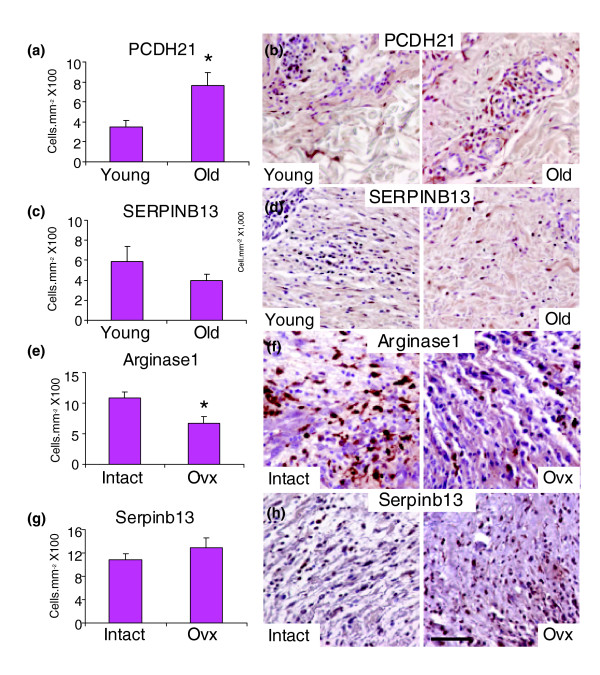
Immunohistochemical analysis of proteins encoded by array-identified dermally expressed genes during human and murine wound healing. **(a-d) **In agreement with array findings, human wound granulation tissue protein levels of Protocadherin 21 (a) and SERPINB13 protein (b) mirrors changes in gene expression. **(e,f) **In mice, the number of granulation tissue cells expressing arginase 1 is reduced in wounds from ovx mice, mirroring the human array findings and validating this gene/protein as estrogen regulated. **(g,h) **In contrast, yet also in agreement with human array findings, the number of granulation tissue cells expressing serpinb13 was not significantly altered between intact and ovx mice, that is, not dependent on estrogen levels. Results are presented as mean ± standard error of the mean; *p < 0.05. The scale bar in (h) represents 45 μm (b,d), 20 μm (f) and 35 μm (h); n = 3-6 per group.

We then turned our attention to expression of proteins encoded by array-identified genes in cells within the granulation tissue of both human and mouse wounds (Figure 7). Protocadherin 21 (*PCDH21*), identified in this study as 12-fold up-regulated at the level of gene expression in wounds from elderly subjects, but belonging to neither age-associated nor estrogen-regulated subsets (Table 3), displayed statistically significant up-regulation in elderly wounds also at the protein level (Figure 7a,b). Notably, *PCDH21 *has not previously been associated with wound healing, aging or estrogen-regulation. Serpin peptidase inhibitor, clade B (ovalbumin), member 13 (*SERPINB13*), identified in this study as age-associated but not estrogen regulated, and 15-fold down-regulated in wounds from elderly subjects at the level of expression, was also reduced in elderly wounds at the protein level (Figure 7c,d).

Another estrogen-regulated gene with a potentially important role in healing is that encoding arginase 1 (*ARG1*; 82-fold down-regulated in wounds from elderly males). We find significantly less *Arg1 *expressing cells in the wound granulation tissue from ovx mice (Figure 7e,f). While *Arg1 *is known to be estrogen regulated in uterus and prostate, it has not previously been shown to be estrogen regulated in skin. Again, this novel finding may be important in light of the role of arginase in modulating the balance between inflammation and matrix deposition during healing, and in the progression of chronic wounds [23]. Finally, we returned to *Serpinb13 *(a gene identified in this study in human as age-associated but not estrogen-regulated) and determined protein levels in wounds from ovx and intact young female mice. Immunohistochemical analysis demonstrated that the number of cells expressing Serpinb13 protein was unaltered by estrogen status in young female mice (Figure 7g,h), validating this gene as age-associated but not estrogen-regulated.

## Conclusion

Our data clearly implicate estrogen, and not candidate gerontogenes nor 'age-associated' genes, as the most potent regulator of age-associated delay in human wound healing, a discovery underscored by the numerous associations between estrogen-regulated gene polymorphisms and phenotypes representing aging phenomena, including wound healing [24,25]. Whilst expression changes in a few genes that appear to be specifically associated with chronological age were noted, the majority of these genes were indeed also estrogen regulated. It is likely, in fact, that there is an intimate relationship between hormones and aging. Recent reports suggest that the model organism *Caenorhabditis elegans *contains several hormonal steroids that can increase lifespan by up to 20%, and that the insulin growth factor/insulin pathway influences the rate of aging [26,27]. That regulation by estrogen at the level of the gene appears to be the most important mediator of age-related delayed wound healing suggests that post-transcriptional aging phenomena such as free radical damage, glycation, and protein error do not play a major role in this process. We suggest that tissue repair acts as a paradigm for the effects of estrogen on other age-related patho-physiological processes, linking estrogen-regulated genes directly to a protective repair/maintenance program and thus abating 'aging'.

## Materials and methods

### Sample collection and histology

Local ethical committee approval was obtained for all human studies. Following informed consent eighteen healthy, young males and nineteen health status-defined elderly males underwent two 4 mm punch biopsies from the left upper inner arm after local infiltration with 1 ml of 1% lignocaine. The first biopsy (normal skin) was followed by re-biopsy of the same site at one of four pre-defined time points to excise wound tissue. The selected time points were three days, seven days, three months and six months post-initial biopsy. Ten-week-old female C57/Bl6 mice with intact ovaries and ten-week-old C57/Bl6 mice that had undergone ovariectomy one month previously were anesthetized and wounded following our established protocol [9] (in accordance with home office regulations). Briefly, two equidistant 1 cm full-thickness skin incisional wounds were made through both skin and panniculus carnosus muscle, and left to heal by secondary intention. Wounds were excised and bisected at day 3 after wounding, with one-half of the sample processed for histology.

Histological sections were prepared from biopsy/mouse wound tissue fixed in 10% buffered formalin and embedded in paraffin. Sections (5 μm) were stained with hematoxylin and eosin, or subjected to immunohistochemistry with mouse monoclonal anti-CD15 (BD Biosciences, Pharmingen, Oxford, UK), mouse monoclonal anti-CD68, anti-VWF (Dako, Cambridge, UK), anti-MIF goat polyclonal antibody (R&D Systems, Abingdon, UK), anti-LOR, anti-INV (Covance, Berkeley, CA, USA), JCMC (rabbit polyclonal anti-Dsc1), anti-K16, anti-ARG1 (Santa Cruz Biotechnology, Santa Cruz, CA, USA), anti-PCDH21 or anti-SERPINB13 (Abcam, Cambridge, UK) and the appropriate biotinylated secondary antibody followed by ABC-peroxidase reagent (Vector Laboratories, Peterborough, UK) with Novared substrate and counterstaining with hematoxylin. Control slides stained with secondary antibody in isolation or control IgG were negative. Total cell numbers and re-epithelialization were quantified with Image Pro Plus software as previously described [8] (MediaCybernetics, Silver Spring, MD, USA).

### Sample collection and RNA preparation

Following informed consent, five healthy, young males (24-27 years old) and five health status-defined elderly males (71-76 years old) underwent two 4 mm punch biopsies from the left upper inner arm after local infiltration with 1 ml of 1% lignocaine. The first biopsy (normal skin) was followed by re-biopsy of the same site seven days later to excise wound tissue. In addition, ten-week-old female BALB/c mice with intact ovaries and ten-week-old mice that had undergone ovariectomy one month previously were anesthetized and wounded following our established protocol [9] (in accordance with home office regulations). Wounds were excised and bisected three days after wounding, and one-half of the wound was flash frozen at -80°C before RNA extraction. Total RNA was isolated from frozen tissue by homogenizing in Trizol reagent (Invitrogen, Paisley, UK) following the manufacturer's instructions.

### Cell culture

Mouse peritoneal macrophages were isolated by intraperitoneal lavage with ice-cold sterile phosphate-buffered saline, pooled for subsequent studies and cell viability determined by trypan blue. Cells were re-suspended at a concentration of 10^6 ^cells per ml in serum-free phenol-red free Dulbecco's modified Eagle's medium (DMEM) medium, treated with lipopolysaccharide (1 mg/ml) and 10^-7 ^or 10^-8^ M 17-β-estradiol (Sigma-Aldrich, St Louis, MO, USA) or lipopolysaccharide alone. Cells were washed, 0.5 ml TRIzol (Invitrogen Corp., Carlsbad, CA, USA) was added per well, and plates were stored at -80°C before RNA extraction. Mouse dermal fibroblasts were isolated as follows. Epidermis and dermis were separated following overnight incubation in 0.25% Trypsin/EDTA (Cascade Biologics, Portland, OR, USA) at 4°C. Minced dermis was incubated in 0.3% collagenase in DMEM for 30 minutes at 37°C in 5% CO_2 _atmosphere with regular agitation. The collagenase-cell mixture was filtered, centrifuged, and isolated fibroblasts washed with fresh media (DMEM, 5% fetal calf serum, L-glutamine, 1% Penicillin, Streptomycin, and Amphotericin B (PSA) prior to plating. Cells were plated and cultured until confluent in DMEM medium supplemented with Penicillin/Streptomycin and 10% charcoal-stripped fetal calf serum (Thermo Scientific, Waltham, MA, USA). Confluent fibroblasts were treated with lipopolysaccharide (1 mg/ml) and 10^-8 ^or 10^-9 ^M 17-β-estradiol (Sigma-Aldrich) or lipopolysaccharide alone. Cells were washed, 0.5 ml TRIzol (Invitrogen Corp.) was added per well, and plates were stored at -80°C before RNA extraction.

### Microarray analysis

Human microarray experiments were performed using the human genome U133A oligonucleotide array (Affymetrix Inc., High Wycombe, UK) according to the manufacturer's instructions. Total wound RNA (100 ng) from three old and three young male subjects was used with the Two-Cycle cDNA Synthesis Kit (P/N 900432 Affymetrix Inc.; one sample hybridized per array). Technical quality control was performed with dChip [28]. Background correction, quantile normalization, and gene expression analysis were performed using GCRMA [29]. The microarray data were submitted in MIAME (minimum information about a microarray experiment)-compliant format to the ArrayExpress database [30]. Differential expression between the young and old groups was tested statistically with CyberT on logarithmic scale data [31]. False-discovery correction was performed with Q-value software [32]. Significantly changed probe sets were selected on fold change (±7-fold), q-value (<0.1) and expression level (>15) (see Additional data file 1 for a full list). For up- and down-regulated gene data sets overrepresented GO groups were identified using the second generation (DAVID 2007) expression analysis systematic explorer (EASE) online functional annotation tool [33] (Additional data file 5). Mouse microarray experiments were performed as previously described [9], ArrayExpress database accession number e-mexp-209. Briefly, biotinylated cRNA samples from individual intact and ovx mice were hybridized to mouse 430A oligonucleotide arrays (Affymetrix Inc., Santa Clara, CA, USA). For this current experiment data were re-analyzed using GCRMA for background correction, quantile normalization, and gene expression analysis [29]. Differential expression between the intact and ovx groups was tested statistically with CyberT on logarithmic scale data [31]. Significantly changed probe sets were selected on fold change (±1.5-fold), *p*-value (<0.1) and expression level (>50).

Hormonally regulated genes were identified by: comparison of significantly changed human probesets (Additional data file 1) with the Dragon online database of estrogen regulated genes [11] to generate conserved gene subset S1 (Additional data file 2); cross-species comparison of human genes identified in this study (U133A microarrays) with genes up- or down-regulated in hormonally mediated delayed-healing murine wounds (430A microarrays) to generate conserved gene subset S2 (Additional data file 3); and hand annotation as estrogen-regulated following an exhaustive literature search to generate conserved gene subset S3 (Additional data file 4). Aging-associated genes were identified using: comparison of significantly changed human probesets (Additional data file 1) with the hand curated GenAge database of aging-associated genes [12] to generate conserved gene subset S4 (Additional data file 6); combined with genes annotated with known age-associated ontology groups (DNA damage, mitochondria, neurodegeneration or response to UV) and/or hand annotated as age-associated following an exhaustive literature search to generate conserved gene subset S5 (Additional data file 7).

### qPCR and comparison with microarrays

cDNA was transcribed from 0.5 μg of human wound RNA (five old and five young male subjects), from 0.5 μg of human normal skin RNA (three old and three young subjects), from 1 μg of mouse wound RNA (six intact and six oxv mice) and 1 μg of RNA isolated from estrogen-treated macrophages or fibrolasts. (Promega RT kit and AMV-reverse transcriptase; Roche, Welwyn Garden City, UK). Primer sequences were designed to each gene coding sequence independently of the Affymetrix probe set target region sequence and hence may or may not be directed to the same gene region. qPCR was performed using the SYBR green core kit (Eurogentec, Southampton, UK) following the manufacturer's instructions and an Opticon qPCR thermal cycler (Bio-Rad, Hemel Hempstead, UK). For each primer set an optimal dilution was determined, and melting curves were used to determine product specificity. Each sample was serially diluted over three orders of magnitude, and for each primer set all samples were run on the same 96-well plate. For primer sequences see Additional data file 9. Expression ratios were determined relative to a standard sample and normalized using a value derived from three separate control primer sets to 18s rRNA and the housekeeping genes *Gapdh *and *Ywahz*. In Figure 4, fold change is presented relative to young normal skin with values below 1 converted to negative fold change. In Figure 5, data for estrogen treated cells is presented as fold change relative to each cell type treated with lipopolysaccharide alone with values below 1 again converted to negative fold change.

### Data deposition

Microarray data have been deposited in MIAME compliant format in the ArrayExpress database [34], accession number E-MEXP-1074.

## Abbreviations

DMEM, Dulbecco's modified Eagle's medium; EASE, expression analysis systematic explorer; GO, gene ontology; MIAME, minimum information about a microarray experiment; ovx, ovariectomised; qPCR, quantitative real-time PCR.

## Authors' contributions

MJH was involved in study design, carried out experiments and data analysis, and was involved in manuscript preparation. GSA was involved in study design and manuscript preparation. Both MJH and GSA have had full access to all of the data in the study and take responsibility for the integrity of the data and the accuracy of the data analysis.

## Additional data files

The following additional data are available. Additional data file 1 is a table listing all probe sets identified as differentially expressed using the filtering criteria fold change (±7-fold), q-value (<0.1) and expression level (>15). Additional data file 2 is a table listing the full subset S1 - Dragon database-derived estrogen-regulated probe sets. Additional data file 3 is a table listing the full subset S2 - mouse dataset-derived estrogen-regulated probe sets. Additional data file 4 is a table listing the full subset S3 - hand-annotated estrogen-regulated probe sets. Additional data file 5 is a table listing the full EASE experimental readout. Additional data file 6 is a table listing the full subset S4 - GenAge-derived aging-associated probe sets. Additional data file 7 is a table listing the full subset S5 - hand-curated and aging-associated GO probe sets. Additional data file 8 is a table listing differentially expressed genes identified in this study, using a relaxed array filtering criteria, that have also been demonstrated to alter life-span in animal models. Additional data file 9 is a table listing all primers used for qPCR.

## Supplementary Material

Additional data file 1 All probe sets identified as differentially expressed using the filtering criteria fold change (±7-fold), q-value (<0.1) and expression level (>15). Click here for file

Additional data file 2 Subset S1: Dragon database-derived estrogen-regulated probe sets. Click here for file

Additional data file 3 Subset S2: mouse dataset-derived estrogen-regulated probe sets. Click here for file

Additional data file 4 Subset S3: hand-annotated estrogen-regulated probe sets. Click here for file

Additional data file 5 EASE experimental readout. Click here for file

Additional data file 6 Subset S4: GenAge-derived aging-associated probe sets. Click here for file

Additional data file 7 Subset S5: hand-curated and aging-associated GO probe sets. Click here for file

Additional data file 8 Differentially expressed genes identified in this study, using a relaxed array filtering criteria, that have also been demonstrated to alter life-span in animal models. Click here for file

Additional data file 9 Primers used for qPCR. Click here for file
