# Supplementary material for: Estrogen, not intrinsic aging, is the major regulator of delayed human wound healing in the elderly
Source: Genome Biol. 2008 May 13;9(5):R80. doi: 10.1186/gb-2008-9-5-r80 (PMC2441466; doi:10.1186/gb-2008-9-5-r80)
Supplement: Additional data file 1 — All probe sets identified as differentially expressed using the filtering criteria fold change (±7-fold), q-value (<0.1) and expression level (>15). [file gb-2008-9-5-r80-S1.doc]

**Supplementary Table 1 – The full list of probe sets identified using the filtering criteria fold change (+/-7 fold), q-value (< 0.1) and expression level (>15) up (green) & down (red**) in old.

| **Affy ID** | **Gene^a^** | **Public ID** | **Function** | **q value^b^** | **FC^c^** |
| --- | --- | --- | --- | --- | --- |
| 207720_at | **LOR** | NM_000427 | Major cornified envelope protein | 0 | -235 |
| 201909_at | RPS4Y1 | NM_001008 | 40S ribosomal component | 0 | -142 |
| 215704_at | FLG | AL356504 | Cornified envelope-keratin linker protein | 9.4E-13 | -114 |
| 206177_s_at | **ARG1** | NM_000045 | Delayed healing-associated | 9.2E-11 | -82.0 |
| 205000_at | **DDX3Y** | NM_004660 | DEAD (Asp-Glu-Ala-Asp) box polypeptide 3... | 5.9E-13 | -78.6 |
| 206643_at | HAL | NM_002108 | Histidine catabolism | 1.5E-10 | -59.0 |
| 206421_s_at | **SERPINB7** | NM_003784 | Proteinase inhibitor for plasmin | 5.9E-13 | -47.6 |
| 206192_at | CDSN | L20815 | Late epidermal terminal differentiation | 2.5E-06 | -31.0 |
| 213796_at | SPRR1A | AI923984 | Cornified envelope precursor protein | 1.5E-05 | -29.4 |
| 207324_s_at | **DSC1** | NM_004948 | Desmosomal cadherin / adhesion | 9.6E-06 | -28.9 |
| 209719_x_at | SERPINB3 | BC005224 | Inflammation and cancer-associated | 1.6E-05 | -22.4 |
| 217496_s_at | **IDE** | AA918442 | Wound fluid / resolution of insulin response | 4.0E-06 | -20.5 |
| 217521_at | --- | N54942 | Unknown | 1.1E-05 | -20.1 |
| 211597_s_at | HOP | AB059408 | Serum response factor binding | 1.7E-04 | -19.7 |
| 211726_s_at | FMO2 | BC005894 | Non-functional oxidative enzyme | 7.0E-04 | -18.9 |
| 220414_at | CALML5 | NM_017422 | Epidermal-associated calcium-binding | 2.0E-05 | -17.7 |
| 203328_x_at | **IDE** | NM_004969 | Wound fluid / resolution of insulin response | 1.4E-05 | -17.4 |
| 210413_x_at | SERPINB4 | U19557 | Cancer and inflammation-associated | 3.1E-05 | -15.8 |
| 219795_at | SLC6A14 | NM_007231 | Amino acid transport / obesity | 6.9E-04 | -15.6 |
| 210074_at | **CTSL2** | AF070448 | Lysosomal cysteine proteinase | 3.8E-05 | -15.5 |
| 217272_s_at | **SERPINB13** | AJ001698 | Keratinocyte expressed cathepsin inhibitor | 1.7E-04 | -15.0 |
| 222242_s_at | KLK5 | AF243527 | Desquamation, angiogenesis & cancer | 4.0E-05 | -15.0 |
| 201348_at | **GPX3** | NM_002084 | Protection from oxidative damage | 1.2E-05 | -14.8 |
| 202018_s_at | LTF | NM_002343 | Inflammatory-cell-derived antioxidant | 4.6E-02 | -14.5 |
| 205185_at | SPINK5 | NM_006846 | Anti-inflammatory/microbial protease inhibitor | 3.8E-05 | -14.4 |
| 213780_at | TCHH | N30878 | Hair-follicle cornified envelope protein | 1.0E-02 | -13.8 |
| 219099_at | C12orf5 | NM_020375 | Unknown | 4.2E-04 | -12.8 |
| 211906_s_at | SERPINB4 | AB046400 | Cancer and inflammation-associated | 5.7E-05 | -12.4 |
| 219232_s_at | EGLN3 | NM_022073 | Hypoxia-inducible apoptosis-inducing protein | 1.4E-05 | -12.1 |
| 213256_at | MARCH3 | AW593996 | Poorly characterized ubiquitin ligase | 1.6E-05 | -12.1 |
| 204733_at | **KLK6** | NM_002774 | Hormone regulated serine protease | 1.4E-05 | -11.9 |
| 202179_at | BLMH | NM_000386 | Cysteine peptidase | 2.1E-03 | -11.8 |
| 214549_x_at | SPRR1A | NM_005987 | Cornified envelope precursor protein | 1.6E-04 | -11.3 |
| 207908_at | KRT2 | NM_000423 | Supra-basally expressed cytokeratin | 1.2E-03 | -11.1 |
| 210338_s_at | HSPA8 | AB034951 | ERalpha-inhibiting heat shock protein | 9.9E-04 | -10.6 |
| 209720_s_at | SERPINB3 | BC005224 | Inflammation and cancer-associated | 3.3E-04 | -10.5 |
| 201849_at | BNIP3 | NM_004052 | Mitochondrial apoptosis inducing protein | 2.7E-04 | -10.1 |
| 205916_at | S100A7 | NM_002963 | Chemotactic psoriasis-associated protein | 1.7E-04 | -10.0 |
| 220322_at | IL1F9 | NM_019618 | Keratinocyte cytokine | 9.7E-04 | -9.9 |
| 218454_at | FLJ22662 | NM_024829 | Unknown | 1.2E-03 | -9.9 |
| 204952_at | LYPD3 | NM_014400 | Upregulated in migrating keratinocytes | 1.2E-03 | -9.7 |
| 206595_at | CST6 | NM_001323 | Cysteine protease inhibitor | 1.7E-06 | -9.3 |
| 203327_at | **IDE** | N22903 | Wound fluid / resolution of insulin response | 7.0E-04 | -9.3 |
| 209555_s_at | **CD36** | BE968792 | Thrombospondin receptor | 4.0E-03 | -9.2 |
| 219532_at | ELOVL4 | NM_022726 | Skin barrier-promoting fatty acid elongase | 1.5E-05 | -9.2 |
| 209126_x_at | KRT6B | L42612 | Injury-associated keratin | 1.7E-03 | -9.1 |
| 212573_at | ENDOD1 | AF131747 | Unknown | 8.3E-04 | -9.0 |
| 218150_at | ARL5A | NM_012097 | Developmentally regulated nuclear protein | 1.8E-03 | -9.0 |
| 214599_at | IVL | NM_005547 | Early cornified envelope protein | 2.8E-03 | -8.8 |
| 209218_at | SQLE | AF098865 | Rate-limiting sterol biosynthesis enzyme | 7.2E-04 | -8.8 |
| 207356_at | DEFB4 | NM_004942 | Antimicrobial peptide | 6.0E-03 | -8.8 |
| 210138_at | RGS20 | AF074979 | TLR regulated GTPase-activating protein | 8.1E-04 | -8.7 |
| 205001_s_at | DDX3Y | AF000985 | Male fertility-associated RNA helicase | 1.1E-05 | -8.6 |
| 202504_at | TRIM29 | NM_012101 | Cancer-associated transcription factor | 2.2E-03 | -8.6 |
| 205016_at | TGFA | NM_003236 | IFN-induced / role in epidermal regeneration | 1.0E-03 | -8.5 |
| 209309_at | AZGP1 | D90427 | TNFA-regulated prostate-cancer marker | 3.5E-04 | -8.5 |
| 209800_at | KRT16 | AF061812 | Hyperproliferation & healing-assoc. keratin | 1.2E-03 | -8.3 |
| 205778_at | KLK7 | NM_005046 | Innate immunity / desquamation | 1.2E-05 | -8.3 |
| 214131_at | CYorf15B | AL049280 | Unknown | 1.1E-03 | -8.1 |
| 219756_s_at | POF1B | NM_024921 | Unknown | 3.9E-05 | -8.1 |
| 214091_s_at | **GPX3** | AW149846 | Protection from oxidative damage | 3.0E-03 | -8.1 |
| 203585_at | ZNF185 | NM_007150 | Actin-associated tumor suppressor | 1.4E-03 | -8.1 |
| 206008_at | TGM1 | NM_000359 | CE formation / Epidermal differentiation | 4.6E-05 | -8.0 |
| 203180_at | ALDH1A3 | NM_000693 | Detoxification of aldehydes | 7.8E-03 | -8.0 |
| 202037_s_at | SFRP1 | AF017987 | Repressor of WNT signalling | 6.6E-04 | -7.9 |
| 207602_at | TMPRSS11D | NM_004262 | Psoriasis-associated serine protease | 2.3E-04 | -7.9 |
| 202539_s_at | HMGCR | NM_000859 | Rate-limiting enzyme for cholesterol synthesis | 7.4E-04 | -7.8 |
| 203575_at | CSNK2A2 | NM_001896 | p53 phosphorylation, WNT signaling pathway | 4.6E-04 | -7.7 |
| 206884_s_at | SCEL | NM_003843 | Cornified envelope precursor protein | 2.1E-04 | -7.5 |
| 204284_at | PPP1R3C | N26005 | Regulates a wide variety of cellular functions | 9.9E-04 | -7.4 |
| 266_s_at | CD24 | L33930 | Molecular marker for epithelial neoplasms | 2.7E-04 | -7.4 |
| 203914_x_at | HPGD | NM_000860 | Main enzyme of prostaglandin degradation | 1.6E-04 | -7.3 |
| 212907_at | **SLC30A1** | AI972416 | Zinc / Calcium ion transporter | 8.5E-04 | -7.3 |
| 219410_at | TMEM45A | NM_018004 | Hox-regulated/reproductive tissue expressed | 8.1E-04 | -7.3 |
| 206488_s_at | **CD36** | NM_000072 | Thrombospondin receptor | 1.2E-05 | -7.3 |
| 204881_s_at | UGCG | NM_003358 | Keratinocyte ceramide glucosyltransferase | 1.8E-03 | -7.1 |
| 213933_at | PTGER3 | AW242315 | Impaired wound healing in null mouse | 8.3E-04 | -7.1 |
| 216379_x_at | CD24 | AK000168 | Molecular marker for epithelial neoplasms | 7.9E-04 | -7.0 |
| 221728_x_at | **XIST** | AA628440 | X chromosome inactivation | 2.4E-12 | 192 |
| 214218_s_at | **XIST** | AV699347 | X chromosome inactivation | 1.0E-09 | 56.2 |
| 213369_at | PCDH21 | AI825832 | Adhesion | 1.3E-05 | 11.9 |
| 221501_x_at | LOC339047 | AF229069 | Unknown | 9.8E-05 | 9.3 |
| 206211_at | **SELE** | NM_000450 | Endothelial-leukocyte adhesion mediator | 9.0E-02 | 8.5 |
| 211600_at | **PTPRO** | U20489 | Newly identified marker of podocyte injury | 5.0E-04 | 8.4 |
| 220940_at | KIAA1641 | NM_025190 | Unknown | 1.0E-04 | 8.3 |
| 203915_at | **CXCL9** | NM_002416 | Interferon induced, TH1 response-associated | 6.3E-02 | 7.3 |
| 204324_s_at | GOLPH4 | NM_014498 | Protein export | 8.3E-04 | 7.3 |
| 201205_at | RRBP1 | AF006751 | Developmentally regulated ECM glycoprotein | 6.3E-03 | 7.3 |

a. Genes in **bold** have been validated by Real-time PCR.

b. CyberT-derived multiple testing corrected q-value

c. Fold change (old/young)
