# Supplementary material for: Estrogen, not intrinsic aging, is the major regulator of delayed human wound healing in the elderly
Source: Genome Biol. 2008 May 13;9(5):R80. doi: 10.1186/gb-2008-9-5-r80 (PMC2441466; doi:10.1186/gb-2008-9-5-r80)
Supplement: Additional data file 2 — Subset S1: Dragon database-derived estrogen-regulated probe sets. [file gb-2008-9-5-r80-S2.doc]

**Supplementary table 2 – Subset 1 (s1): Dragon database probe sets that are differentially expressed in wounds from young and elderly subjects, up (green) & down (red**) in old.

| **Affy ID** | **Gene^a^** | **Gene (Description)** | **Function** | **q value^b^** | **FC^c^** |
| --- | --- | --- | --- | --- | --- |
| 206177_s_at | **ARG1** | arginase, liver | Delayed healing-associated | 9.2E-11 | -82.0 |
| 222242_s_at | KLK5 | kallikrein 5 | Desquamation, angiogenesis & cancer | 4.0E-05 | -15.0 |
| 202018_s_at | LTF | lactotransferrin | Inflammatory-cell-derived antioxidant | 4.6E-02 | -14.5 |
| 205185_at | SPINK5 | serine peptidase inhibitor, Kazal type 5 | Anti-inflammatory/microbial protease inhibitor | 3.8E-05 | -14.4 |
| 204733_at | **KLK6** | kallikrein 6 (neurosin, zyme) | Hormone regulated serine protease | 1.4E-05 | -11.9 |
| 210338_s_at | **HSPA8** | heat shock 70kDa protein 8 | ERalpha-inhibiting heat shock protein | 9.9E-04 | -10.6 |
| 201849_at | **BNIP3** | BCL2/adenovirus E1B 19kDa interacting... | Mitochondrial apoptosis inducing protein | 2.7E-04 | -10.1 |
| 209126_x_at | KRT6B | keratin 6B | Injury-associated keratin | 1.7E-03 | -9.1 |
| 209218_at | SQLE | squalene epoxidase | Rate-limiting sterol biosynthesis enzyme | 7.2E-04 | -8.8 |
| 207356_at | DEFB4 | defensin, beta 4 | Antimicrobial peptide | 6.0E-03 | -8.8 |
| 205016_at | TGFA | transforming growth factor, alpha | IFN-induced / role in epidermal regeneration | 1.0E-03 | -8.5 |
| 205778_at | KLK7 | kallikrein 7 (chymotryptic, stratum corne... | Innate immunity / desquamation | 1.2E-05 | -8.3 |
| 202037_s_at | SFRP1 | secreted frizzled-related protein 1 | Repressor of WNT signalling | 6.6E-04 | -7.9 |
| 202539_s_at | HMGCR | 3-hydroxy-3-methylglutaryl-Coenzyme… | Rate-limiting cholesterol synthesis enzyme | 7.4E-04 | -7.8 |
| 266_s_at | CD24 | CD24 molecule | Molecular marker for epithelial neoplasms | 2.7E-04 | -7.4 |
| 203914_x_at | HPGD | hydroxyprostaglandin dehydrogenase… | Main enzyme for prostaglandin degradation | 1.6E-04 | -7.3 |
| 204881_s_at | UGCG | UDP-glucose ceramide glucosyltransferase | Keratinocyte ceramide glucosyltransferase | 1.8E-03 | -7.1 |
| 213933_at | PTGER3 | prostaglandin E receptor 3 (subtype EP3) | Impaired wound healing in null mouse | 8.3E-04 | -7.1 |
| 216379_x_at | CD24 | CD24 molecule | Molecular marker for epithelial neoplasms | 7.9E-04 | -7.0 |
| 211600_at | **PTPRO** | protein tyrosine phosphatase, receptor typ… | Newly identified marker of podocyte injury | 5.0E-04 | 8.4 |

a. Genes in **bold** have been validated by Real-time PCR.

b. CyberT-derived multiple testing corrected q-value

c. Fold change (old/young)
