# Supplementary material for: Estrogen, not intrinsic aging, is the major regulator of delayed human wound healing in the elderly
Source: Genome Biol. 2008 May 13;9(5):R80. doi: 10.1186/gb-2008-9-5-r80 (PMC2441466; doi:10.1186/gb-2008-9-5-r80)
Supplement: Additional data file 3 — Subset S2: mouse dataset-derived estrogen-regulated probe sets. [file gb-2008-9-5-r80-S3.doc]

**Supplementary table 3 – Subset 2 (s2): Mouse dataset-derived estrogen-regulated probe sets that are differentially expressed in wounds from young and elderly subjects, up (green) & down (red**) in old.

| **Affy ID** | **Gene^a^** | **Gene (Description)** | **Function** | **q value^b^** | **FC^c^** |
| --- | --- | --- | --- | --- | --- |
| 206192_at | CDSN | Corneodesmosin | Desquamation / Adhesion | 2.51E-06 | -31.0 |
| 213796_at | SPRR1A | small proline-rich protein 1A | Cornified envelope precursor protein | 1.5E-05 | -29.4 |
| 211597_s_at | HOP | homeodomain-only protein | Serum response factor binding | 1.7E-04 | -19.7 |
| 214091_s_at | **GPX3** | glutathione peroxidase 3 (plasma) | Protection from oxidative damage | 1.2E-05 | -14.8 |
| 204733_at | **KLK6** | kallikrein 6 (neurosin, zyme) | Hormone regulated serine protease | 1.4E-05 | -11.9 |
| 214549_x_at | SPRR1A | small proline-rich protein 1A | Cornified envelope precursor protein | 1.6E-04 | -11.3 |
| 204952_at | LYPD3 | LY6/PLAUR domain containing 3 | Upregulated in migrating keratinocytes | 1.2E-03 | -9.7 |
| 209555_s_at | **CD36** | CD36 molecule | Thrombospondin receptor | 4.0E-03 | -9.2 |
| 219532_at | ELOVL4 | elongation of very long chain fatty acids… | Skin barrier-promoting fatty acid elongase | 1.5E-05 | -9.2 |
| 212573_at | ENDOD1 | endonuclease domain containing 1 | Unknown | 8.3E-04 | -9.0 |
| 205778_at | KLK7 | kallikrein 7 (chymotryptic, stratum corneum) | Innate immunity / desquamation | 1.2E-05 | -8.3 |
| 219756_s_at | POF1B | premature ovarian failure, 1B | Unknown | 3.9E-05 | -8.1 |
| 214091_s_at | **GPX3** | glutathione peroxidase 3 (plasma) | Protection from oxidative damage | 3.0E-03 | -8.1 |
| 203585_at | ZNF185 | zinc finger protein 185 (LIM domain) | Actin-associated tumor suppressor | 1.4E-03 | -8.1 |
| 206008_at | TGM1 | transglutaminase 1 | CE formation / Epidermal differentiation | 4.6E-05 | -8.0 |
| 206884_s_at | SCEL | sciellin | Cornified envelope precursor protein | 2.1E-04 | -7.5 |
| 204284_at | PPP1R3C | protein phosphatase 1, regulatory (inhibit… | Regulates a wide variety of cellular functions | 9.9E-04 | -7.4 |
| 219410_at | TMEM45A | transmembrane protein 45A | Hox-regulated/reproductive tissue expressed | 8.1E-04 | -7.3 |
| 206488_s_at | **CD36** | CD36 molecule | Thrombospondin receptor | 1.2E-05 | -7.3 |

a. Genes in **bold** have been validated by Real-time PCR.

b. CyberT-derived multiple testing corrected q-value

c. Fold change (old/young)
