# Supplementary material for: Estrogen, not intrinsic aging, is the major regulator of delayed human wound healing in the elderly
Source: Genome Biol. 2008 May 13;9(5):R80. doi: 10.1186/gb-2008-9-5-r80 (PMC2441466; doi:10.1186/gb-2008-9-5-r80)
Supplement: Additional data file 4 — Subset S3: hand-annotated estrogen-regulated probe sets. [file gb-2008-9-5-r80-S4.doc]

**Supplementary table 4 – Subset 3 (s3): Hand-annotated estrogen-regulated probe sets that are differentially expressed in wounds from young and elderly subjects, up (green) & down (red**) in old.

| **Affy ID** | **Gene^a^** | **Gene (Description)** | **Function** | **q value^b^** | **FC^c^** | **Ref** |
| --- | --- | --- | --- | --- | --- | --- |
| 207720_at | **LOR** | loricrin | Major cornified envelope protein | 0 | -235 | Lin et al., 2004 |
| 201909_at | RPS4Y1 | ribosomal protein S4, Y-linked 1 | 40S ribosomal component | 0 | -142 | Denger et al., 2007 |
| 215704_at | FLG | filaggrin | Cornified envelope-keratin linker protein | 9.4E-13 | -114 | Komuves et al., 1998 |
| 206643_at | HAL | histidine ammonia-lyase | Histidine catabolism | 1.5E-10 | -59.0 | Lamartiniere et al., 1977 |
| 206421_s_at | **SERPINB7** | serpin peptidase inhibitor, clad… | Proteinase inhibitor for plasmin | 5.9E-13 | -47.6 | Hardman et al., 2005 |
| 207324_s_at | **DSC1** | desmocollin 1 | Desmosomal cadherin / adhesion | 9.6E-06 | -28.9 | Acevedo et al., 2004 |
| 209719_x_at | SERPINB3 | serpin peptidase inhibitor, clad… | Inflammation and cancer-associated | 1.6E-05 | -22.4 | Rae et al., 2005 |
| 217496_s_at | **IDE** | insulin-degrading enzyme | Wound fluid / resolution of insulin response | 4.0E-06 | -20.5 | Udrisar et al., 2005 |
| 211726_s_at | FMO2 | flavin containing monooxygen… | Non-functional oxidative enzyme | 7.0E-04 | -18.9 | Denger et al., 2007 |
| 220414_at | CALML5 | calmodulin-like 5 | Epidermal-associated calcium-binding | 2.0E-05 | -17.7 | Denger et al., 2007 |
| 203328_x_at | **IDE** | insulin-degrading enzyme | Wound fluid / resolution of insulin response | 1.4E-05 | -17.4 | Udrisar et al., 2005 |
| 210413_x_at | SERPINB4 | serpin peptidase inhibitor, clad… | Cancer and inflammation-associated | 3.1E-05 | -15.8 | Coser et al., 2003 |
| 219795_at | SLC6A14 | solute carrier family 6 (amino… | Amino acid transport / obesity | 6.9E-04 | -15.6 | li et al., 2006 |
| 210074_at | **CTSL2** | cathepsin L2 | Lysosomal cysteine proteinase | 3.8E-05 | -15.5 | Coser et al., 2003 |
| 211906_s_at | SERPINB4 | serpin peptidase inhibitor, clad… | Cancer and inflammation-associated | 5.7E-05 | -12.4 | Coser et al., 2003 |
| 219232_s_at | EGLN3 | egl nine homolog 3 (C. elegans) | Hypoxia-inducible apoptosis-inducing protein | 1.4E-05 | -12.1 | Lian et al., 2006 |
| 213256_at | MARCH3 | membrane-associated ring fin… | Poorly characterized ubiquitin ligase | 1.6E-05 | -12.1 | Coser et al., 2003 |
| 202179_at | BLMH | bleomycin hydrolase | Cysteine peptidase | 2.1E-03 | -11.8 | Monroe et al., 2005 |
| 207908_at | KRT2 | keratin 2 (epidermal ichthyosis… | Supra-basally expressed cytokeratin | 1.2E-03 | -11.1 | Denger et al., 2007 |
| 209720_s_at | SERPINB3 | serpin peptidase inhibitor, clad… | Inflammation and cancer-associated | 3.3E-04 | -10.5 | Rae et al., 2005 |
| 205916_at | S100A7 | S100 calcium binding protein… | Chemotactic psoriasis-associated protein | 1.7E-04 | -10.0 | Skliris et al., 2007 |
| 206595_at | CST6 | cystatin E/M | Cysteine protease inhibitor | 1.7E-06 | -9.3 | Coser et al., 2003 |
| 203327_at | **IDE** | insulin-degrading enzyme | Wound fluid / resolution of insulin response | 7.0E-04 | -9.3 | Udrisar et al., 2005 |
| 214599_at | IVL | Involucrin | Early cornified envelope protein | 2.8E-03 | -8.8 | Kurita et al., 2001 |
| 210138_at | RGS20 | regulator of G-protein signallin… | TLR regulated GTPase-activating protein | 8.1E-04 | -8.7 | Hardman et al., 2005 |
| 202504_at | TRIM29 | tripartite motif-containing 29 | Cancer-associated transcription factor | 2.2E-03 | -8.6 | Hardman et al., 2005 |
| 209309_at | AZGP1 | alpha-2-glycoprotein 1, zinc | TNFA-regulated prostate-cancer marker | 3.5E-04 | -8.5 | Denger et al., 2007 |
| 209800_at | KRT16 | keratin 16 (focal non-epidermo… | Hyperproliferation & healing-assoc. keratin | 1.2E-03 | -8.3 | Terasaka et al., 2004 |
| 203575_at | CSNK2A2 | casein kinase 2, alpha prime… | p53 phosphorylation, WNT signaling pathway | 4.6E-04 | -7.7 | Coser et al., 2003 |
| 221728_x_at | **XIST** | X (inactive)-specific transcript | X chromosome inactivation | 2.4E-12 | 192 | Denger et al., 2007 |
| 214218_s_at | **XIST** | X (inactive)-specific transcript | X chromosome inactivation | 1.0E-09 | 56.2 | Denger et al., 2007 |
| 206211_at | **SELE** | selectin E (endothelial adhesio… | Endothelial-leukocyte adhesion mediator | 9.0E-02 | 8.5 | Shumino et al., 2005 |
| 220940_at | KIAA1641 | KIAA1641 | Unknown | 1.0E-04 | 8.3 | Denger et al., 2007 |
| 203915_at | **CXCL9** | chemokine (C-X-C motif) ligand.. | Interferon induced, TH1 response-associated | 6.3E-02 | 7.3 | Garidou et al., 2004 |
| 204324_s_at | GOLPH4 | golgi phosphoprotein 4 | Protein export | 8.3E-04 | 7.3 | Moggs et al., 2004 |
| 201205_at | RRBP1 | ribosome binding protein 1 ho… | Developmentally regulated ECM glycoprotein | 6.3E-03 | 7.3 | Moggs et al., 2004 |

a. Genes in **bold** have been validated by Real-time PCR.

b. CyberT-derived multiple testing corrected q-value

c. Fold change (old/young)
