# Supplementary material for: Estrogen, not intrinsic aging, is the major regulator of delayed human wound healing in the elderly
Source: Genome Biol. 2008 May 13;9(5):R80. doi: 10.1186/gb-2008-9-5-r80 (PMC2441466; doi:10.1186/gb-2008-9-5-r80)
Supplement: Additional data file 5 — EASE experimental readout. [file gb-2008-9-5-r80-S5.doc]

**Supplementary Table 5 – Full EASE readout. Overrepresented gene ontology groups**

| **Estrogen regulated and down in elderly** | | | | |
| --- | --- | --- | --- | --- |
| *Category* | *Term* | *Count* | *%* | *P-value* |
| GOTERM_BP_ALL | [ectoderm development](http://www.ebi.ac.uk/ego/GSearch?query=ectoderm development&mode=name) | 14 | 25.5 | 3.1E-17 |
| GOTERM_BP_ALL | [epidermis development](http://www.ebi.ac.uk/ego/GSearch?query=epidermis development&mode=name) | 13 | 23.6 | 2.7E-16 |
| GOTERM_BP_ALL | [tissue development](http://www.ebi.ac.uk/ego/GSearch?query=tissue development&mode=name) | 15 | 27.3 | 1E-14 |
| GOTERM_BP_ALL | [epidermal cell differentiation](http://www.ebi.ac.uk/ego/GSearch?query=epidermal cell differentiation&mode=name) | 5 | 9.1 | 5.1E-07 |
| GOTERM_BP_ALL | [development](http://www.ebi.ac.uk/ego/GSearch?query=development&mode=name) | 23 | 41.8 | 5.5E-07 |
| GOTERM_BP_ALL | [epidermis morphogenesis](http://www.ebi.ac.uk/ego/GSearch?query=epidermis morphogenesis&mode=name) | 5 | 9.1 | 6.8E-07 |
| GOTERM_BP_ALL | [tissue morphogenesis](http://www.ebi.ac.uk/ego/GSearch?query=tissue morphogenesis&mode=name) | 5 | 9.1 | 2.7E-06 |
| SP_PIR_KEYWORDS | [keratinization](http://us.expasy.org/cgi-bin/get-entries?KW=keratinization) | 4 | 7.3 | 0.000017 |
| GOTERM_BP_ALL | [keratinocyte differentiation](http://www.ebi.ac.uk/ego/GSearch?query=keratinocyte differentiation&mode=name) | 4 | 7.3 | 0.000019 |
| GOTERM_BP_ALL | [keratinization](http://www.ebi.ac.uk/ego/GSearch?query=keratinization&mode=name) | 4 | 7.3 | 0.000025 |
| GOTERM_BP_ALL | [cell differentiation](http://www.ebi.ac.uk/ego/GSearch?query=cell differentiation&mode=name) | 11 | 20 | 0.000026 |
| SP_PIR_KEYWORDS | [epidermis](http://us.expasy.org/cgi-bin/get-entries?KW=epidermis) | 3 | 5.5 | 0.00013 |
| GOTERM_CC_ALL | [cornified envelope](http://www.ebi.ac.uk/ego/GSearch?query=cornified envelope&mode=name) | 3 | 5.5 | 0.00027 |
| SP_PIR_KEYWORDS | [direct protein sequencing](http://us.expasy.org/cgi-bin/get-entries?KW=direct protein sequencing) | 20 | 36.4 | 0.00066 |
| KEGG_PATHWAY | [CELL COMMUNICATION](http://david.abcc.ncifcrf.gov/kegg.jsp?PATH=HSA01430$CELL COMMUNICATION) | 5 | 9.1 | 0.0027 |
| SP_PIR_KEYWORDS | [protease inhibitor](http://us.expasy.org/cgi-bin/get-entries?KW=protease inhibitor) | 4 | 7.3 | 0.0038 |
| GOTERM_CC_ALL | [cell fraction](http://www.ebi.ac.uk/ego/GSearch?query=cell fraction&mode=name) | 10 | 18.2 | 0.0041 |
| GOTERM_CC_ALL | [intermediate filament cytoskeleton](http://www.ebi.ac.uk/ego/GSearch?query=intermediate filament cytoskeleton&mode=name) | 4 | 7.3 | 0.0043 |
| GOTERM_CC_ALL | [intermediate filament](http://www.ebi.ac.uk/ego/GSearch?query=intermediate filament&mode=name) | 4 | 7.3 | 0.0043 |
| GOTERM_CC_ALL | [extracellular region](http://www.ebi.ac.uk/ego/GSearch?query=extracellular region&mode=name) | 11 | 20 | 0.0054 |
| GOTERM_MF_ALL | [structural molecule activity](http://www.ebi.ac.uk/ego/GSearch?query=structural molecule activity&mode=name) | 9 | 16.4 | 0.0063 |
| GOTERM_MF_ALL | [structural constituent of cytoskeleton](http://www.ebi.ac.uk/ego/GSearch?query=structural constituent of cytoskeleton&mode=name) | 4 | 7.3 | 0.007 |
| SP_PIR_KEYWORDS | [cornified cell envelope](http://us.expasy.org/cgi-bin/get-entries?KW=cornified cell envelope) | 2 | 3.6 | 0.0096 |
| GOTERM_MF_ALL | [endopeptidase inhibitor activity](http://www.ebi.ac.uk/ego/GSearch?query=endopeptidase inhibitor activity&mode=name) | 4 | 7.3 | 0.011 |
| GOTERM_MF_ALL | [protease inhibitor activity](http://www.ebi.ac.uk/ego/GSearch?query=protease inhibitor activity&mode=name) | 4 | 7.3 | 0.011 |
| GOTERM_MF_ALL | [endopeptidase activity](http://www.ebi.ac.uk/ego/GSearch?query=endopeptidase activity&mode=name) | 6 | 10.9 | 0.012 |
| GOTERM_MF_ALL | [peptidase activity](http://www.ebi.ac.uk/ego/GSearch?query=peptidase activity&mode=name) | 7 | 12.7 | 0.012 |
| PIR_SUPERFAMILY_NAME | SF002253:loricrin | 2 | 3.6 | 0.014 |
| UP_SEQ_FEATURE | site:Reactive bond | 3 | 5.5 | 0.014 |
| GOTERM_MF_ALL | [serine-type endopeptidase activity](http://www.ebi.ac.uk/ego/GSearch?query=serine-type endopeptidase activity&mode=name) | 4 | 7.3 | 0.016 |
| SP_PIR_KEYWORDS | [keratin](http://us.expasy.org/cgi-bin/get-entries?KW=keratin) | 3 | 5.5 | 0.02 |
| GOTERM_MF_ALL | [serine-type peptidase activity](http://www.ebi.ac.uk/ego/GSearch?query=serine-type peptidase activity&mode=name) | 4 | 7.3 | 0.021 |
| PIR_SUPERFAMILY_NAME | SF002282:cytoskeletal keratin | 3 | 5.5 | 0.021 |
| GOTERM_CC_ALL | [cytoskeleton](http://www.ebi.ac.uk/ego/GSearch?query=cytoskeleton&mode=name) | 8 | 14.5 | 0.022 |
| SP_PIR_KEYWORDS | [lipoprotein](http://us.expasy.org/cgi-bin/get-entries?KW=lipoprotein) | 7 | 12.7 | 0.022 |
| SP_PIR_KEYWORDS | [protease](http://us.expasy.org/cgi-bin/get-entries?KW=protease) | 6 | 10.9 | 0.023 |
| INTERPRO_NAME | [Intermediate filament protein](http://www.ebi.ac.uk/interpro/DisplayIproEntry?ac=IPR001664) | 3 | 5.5 | 0.024 |
| UP_SEQ_FEATURE | active site:Charge relay system | 4 | 7.3 | 0.025 |
| GOTERM_BP_ALL | [lipid metabolism](http://www.ebi.ac.uk/ego/GSearch?query=lipid metabolism&mode=name) | 7 | 12.7 | 0.026 |
| SP_PIR_KEYWORDS | [serine protease inhibitor](http://us.expasy.org/cgi-bin/get-entries?KW=serine protease inhibitor) | 3 | 5.5 | 0.026 |
| SP_PIR_KEYWORDS | [intermediate filament](http://us.expasy.org/cgi-bin/get-entries?KW=intermediate filament) | 3 | 5.5 | 0.028 |
| GOTERM_BP_ALL | [carboxylic acid metabolism](http://www.ebi.ac.uk/ego/GSearch?query=carboxylic acid metabolism&mode=name) | 6 | 10.9 | 0.034 |
| GOTERM_MF_ALL | [serine-type endopeptidase inhibitor activity](http://www.ebi.ac.uk/ego/GSearch?query=serine-type endopeptidase inhibitor activity&mode=name) | 3 | 5.5 | 0.034 |
| GOTERM_BP_ALL | [organic acid metabolism](http://www.ebi.ac.uk/ego/GSearch?query=organic acid metabolism&mode=name) | 6 | 10.9 | 0.034 |
| GOTERM_BP_ALL | [cellular lipid metabolism](http://www.ebi.ac.uk/ego/GSearch?query=cellular lipid metabolism&mode=name) | 6 | 10.9 | 0.035 |
| SMART_NAME | [Tryp_SPc](http://smart.embl.de/smart/do_annotation.pl?DOMAIN=SM00020) | 3 | 5.5 | 0.038 |
| SP_PIR_KEYWORDS | [palmitate](http://us.expasy.org/cgi-bin/get-entries?KW=palmitate) | 4 | 7.3 | 0.04 |
| GOTERM_CC_ALL | [cytosol](http://www.ebi.ac.uk/ego/GSearch?query=cytosol&mode=name) | 5 | 9.1 | 0.042 |
| GOTERM_CC_ALL | [endoplasmic reticulum membrane](http://www.ebi.ac.uk/ego/GSearch?query=endoplasmic reticulum membrane&mode=name) | 3 | 5.5 | 0.047 |
| SP_PIR_KEYWORDS | [tandem repeat](http://us.expasy.org/cgi-bin/get-entries?KW=tandem repeat) | 3 | 5.5 | 0.049 |
| SP_PIR_KEYWORDS | [oxidoreductase](http://us.expasy.org/cgi-bin/get-entries?KW=oxidoreductase) | 6 | 10.9 | 0.049 |
| GOTERM_BP_ALL | [lipid biosynthesis](http://www.ebi.ac.uk/ego/GSearch?query=lipid biosynthesis&mode=name) | 4 | 7.3 | 0.051 |
| GOTERM_CC_ALL | [nuclear envelope-endoplasmic reticulum network](http://www.ebi.ac.uk/ego/GSearch?query=nuclear envelope-endoplasmic reticulum network&mode=name) | 3 | 5.5 | 0.052 |
| INTERPRO_NAME | [Peptidase S1A, chymotrypsin](http://www.ebi.ac.uk/interpro/DisplayIproEntry?ac=IPR001314) | 3 | 5.5 | 0.054 |
| UP_SEQ_FEATURE | repeat:6 | 3 | 5.5 | 0.055 |
| GOTERM_MF_ALL | [enzyme inhibitor activity](http://www.ebi.ac.uk/ego/GSearch?query=enzyme inhibitor activity&mode=name) | 4 | 7.3 | 0.056 |
| INTERPRO_NAME | [Peptidase S1 and S6, chymotrypsin/Hap](http://www.ebi.ac.uk/interpro/DisplayIproEntry?ac=IPR001254) | 3 | 5.5 | 0.061 |
| UP_SEQ_FEATURE | domain:Peptidase S1 | 3 | 5.5 | 0.067 |
| GOTERM_BP_ALL | [proteolysis](http://www.ebi.ac.uk/ego/GSearch?query=proteolysis&mode=name) | 6 | 10.9 | 0.07 |
| UP_SEQ_FEATURE | repeat:5 | 3 | 5.5 | 0.07 |
| INTERPRO_NAME | [Type II keratin](http://www.ebi.ac.uk/interpro/DisplayIproEntry?ac=IPR003054) | 2 | 3.6 | 0.073 |
| GOTERM_MF_ALL | [hydrolase activity](http://www.ebi.ac.uk/ego/GSearch?query=hydrolase activity&mode=name) | 12 | 21.8 | 0.078 |
| GOTERM_MF_ALL | [oxidoreductase activity](http://www.ebi.ac.uk/ego/GSearch?query=oxidoreductase activity&mode=name) | 6 | 10.9 | 0.08 |
| SP_PIR_KEYWORDS | [serine protease](http://us.expasy.org/cgi-bin/get-entries?KW=serine protease) | 3 | 5.5 | 0.082 |
| INTERPRO_NAME | [Calcium-binding protein, S-100/ICaBP type](http://www.ebi.ac.uk/interpro/DisplayIproEntry?ac=IPR001751) | 2 | 3.6 | 0.089 |
| UP_SEQ_FEATURE | repeat:4 | 3 | 5.5 | 0.092 |
| SP_PIR_KEYWORDS | [signal](http://us.expasy.org/cgi-bin/get-entries?KW=signal) | 15 | 27.3 | 0.092 |
| GOTERM_CC_ALL | [membrane fraction](http://www.ebi.ac.uk/ego/GSearch?query=membrane fraction&mode=name) | 6 | 10.9 | 0.092 |
| KEGG_PATHWAY | [BIOSYNTHESIS OF STEROIDS](http://david.abcc.ncifcrf.gov/kegg.jsp?PATH=HSA00100$BIOSYNTHESIS OF STEROIDS) | 2 | 3.6 | 0.092 |
| GOTERM_MF_ALL | [catalytic activity](http://www.ebi.ac.uk/ego/GSearch?query=catalytic activity&mode=name) | 23 | 41.8 | 0.097 |
| SP_PIR_KEYWORDS | [calcium binding](http://us.expasy.org/cgi-bin/get-entries?KW=calcium binding) | 3 | 5.5 | 0.098 |
| **Estrogen regulated and up in elderly** | | | | |
| [*Category*](http://david.abcc.ncifcrf.gov/chartReport.jsp?rowids=&cbBenjamini=true&numRecords=1000&visited=yes&currentList=0&ease=0.1&d-16544-p=1&d-16544-s=1&annot=%2CGOTERM_BP_ALL%2CGOTERM_CC_ALL%2CGOTERM_MF_ALL%2CINTERPRO_NAME%2CPIR_SUPERFAMILY_NAME%2CSMART_NAME%2CBBID%2CBIOCARTA%2CKEGG_PATHWAY%2CCOG_KOG_ONTOLOGY%2CSP_PIR_KEYWORDS%2CUP_SEQ_FEATURE%2CGENETIC_ASSOCIATION_DB&count=1&d-16544-o=2&heading=) | *Term* | [*Count*](http://david.abcc.ncifcrf.gov/chartReport.jsp?rowids=&cbBenjamini=true&numRecords=1000&visited=yes&currentList=0&ease=0.1&d-16544-p=1&d-16544-s=5&annot=%2CGOTERM_BP_ALL%2CGOTERM_CC_ALL%2CGOTERM_MF_ALL%2CINTERPRO_NAME%2CPIR_SUPERFAMILY_NAME%2CSMART_NAME%2CBBID%2CBIOCARTA%2CKEGG_PATHWAY%2CCOG_KOG_ONTOLOGY%2CSP_PIR_KEYWORDS%2CUP_SEQ_FEATURE%2CGENETIC_ASSOCIATION_DB&count=1&d-16544-o=1&heading=) | [*%*](http://david.abcc.ncifcrf.gov/chartReport.jsp?rowids=&cbBenjamini=true&numRecords=1000&visited=yes&currentList=0&ease=0.1&d-16544-p=1&d-16544-s=6&annot=%2CGOTERM_BP_ALL%2CGOTERM_CC_ALL%2CGOTERM_MF_ALL%2CINTERPRO_NAME%2CPIR_SUPERFAMILY_NAME%2CSMART_NAME%2CBBID%2CBIOCARTA%2CKEGG_PATHWAY%2CCOG_KOG_ONTOLOGY%2CSP_PIR_KEYWORDS%2CUP_SEQ_FEATURE%2CGENETIC_ASSOCIATION_DB&count=1&d-16544-o=1&heading=) | [*P-Value*](http://david.abcc.ncifcrf.gov/chartReport.jsp?rowids=&cbBenjamini=true&numRecords=1000&visited=yes&currentList=0&ease=0.1&d-16544-p=1&d-16544-s=7&annot=%2CGOTERM_BP_ALL%2CGOTERM_CC_ALL%2CGOTERM_MF_ALL%2CINTERPRO_NAME%2CPIR_SUPERFAMILY_NAME%2CSMART_NAME%2CBBID%2CBIOCARTA%2CKEGG_PATHWAY%2CCOG_KOG_ONTOLOGY%2CSP_PIR_KEYWORDS%2CUP_SEQ_FEATURE%2CGENETIC_ASSOCIATION_DB&count=1&d-16544-o=1&heading=) |
| GOTERM_BP_ALL | [inflammatory response](http://www.ebi.ac.uk/ego/GSearch?query=inflammatory response&mode=name) | 2 | 28.6 | 0.056 |
| GOTERM_CC_ALL | [integral to membrane](http://www.ebi.ac.uk/ego/GSearch?query=integral to membrane&mode=name) | 4 | 57.1 | 0.087 |
| GOTERM_CC_ALL | [intrinsic to membrane](http://www.ebi.ac.uk/ego/GSearch?query=intrinsic to membrane&mode=name) | 4 | 57.1 | 0.088 |
| SP_PIR_KEYWORDS | [signal](http://us.expasy.org/cgi-bin/get-entries?KW=signal) | 3 | 42.9 | 0.092 |
| GOTERM_MF_ALL | [signal transducer activity](http://www.ebi.ac.uk/ego/GSearch?query=signal transducer activity&mode=name) | 3 | 42.9 | 0.097 |
| **Age-associated and down in elderly** | | | | |
| [*Category*](http://david.abcc.ncifcrf.gov/chartReport.jsp?rowids=&cbBenjamini=true&numRecords=1000&visited=yes&currentList=1&ease=0.1&d-16544-p=1&d-16544-s=1&count=1&annot=%2CGOTERM_BP_ALL%2CGOTERM_CC_ALL%2CGOTERM_MF_ALL%2CINTERPRO_NAME%2CPIR_SUPERFAMILY_NAME%2CSMART_NAME%2CKEGG_PATHWAY%2CCOG_KOG_ONTOLOGY%2CSP_PIR_KEYWORDS%2CUP_SEQ_FEATURE%2CGENETIC_ASSOCIATION_DB&d-16544-o=2&heading=) | *Term* | [*Count*](http://david.abcc.ncifcrf.gov/chartReport.jsp?rowids=&cbBenjamini=true&numRecords=1000&visited=yes&currentList=1&ease=0.1&d-16544-p=1&d-16544-s=5&count=1&annot=%2CGOTERM_BP_ALL%2CGOTERM_CC_ALL%2CGOTERM_MF_ALL%2CINTERPRO_NAME%2CPIR_SUPERFAMILY_NAME%2CSMART_NAME%2CKEGG_PATHWAY%2CCOG_KOG_ONTOLOGY%2CSP_PIR_KEYWORDS%2CUP_SEQ_FEATURE%2CGENETIC_ASSOCIATION_DB&d-16544-o=1&heading=) | [*%*](http://david.abcc.ncifcrf.gov/chartReport.jsp?rowids=&cbBenjamini=true&numRecords=1000&visited=yes&currentList=1&ease=0.1&d-16544-p=1&d-16544-s=6&count=1&annot=%2CGOTERM_BP_ALL%2CGOTERM_CC_ALL%2CGOTERM_MF_ALL%2CINTERPRO_NAME%2CPIR_SUPERFAMILY_NAME%2CSMART_NAME%2CKEGG_PATHWAY%2CCOG_KOG_ONTOLOGY%2CSP_PIR_KEYWORDS%2CUP_SEQ_FEATURE%2CGENETIC_ASSOCIATION_DB&d-16544-o=1&heading=) | [*P-Value*](http://david.abcc.ncifcrf.gov/chartReport.jsp?rowids=&cbBenjamini=true&numRecords=1000&visited=yes&currentList=1&ease=0.1&d-16544-p=1&d-16544-s=7&count=1&annot=%2CGOTERM_BP_ALL%2CGOTERM_CC_ALL%2CGOTERM_MF_ALL%2CINTERPRO_NAME%2CPIR_SUPERFAMILY_NAME%2CSMART_NAME%2CKEGG_PATHWAY%2CCOG_KOG_ONTOLOGY%2CSP_PIR_KEYWORDS%2CUP_SEQ_FEATURE%2CGENETIC_ASSOCIATION_DB&d-16544-o=1&heading=) |
| GOTERM_MF_ALL | [endopeptidase activity](http://www.ebi.ac.uk/ego/GSearch?query=endopeptidase activity&mode=name) | 4 | 44.4 | 0.0008 |
| SP_PIR_KEYWORDS | [protease](http://us.expasy.org/cgi-bin/get-entries?KW=protease) | 4 | 44.4 | 0.00096 |
| GOTERM_MF_ALL | [peptidase activity](http://www.ebi.ac.uk/ego/GSearch?query=peptidase activity&mode=name) | 4 | 44.4 | 0.0022 |
| GOTERM_BP_ALL | [proteolysis](http://www.ebi.ac.uk/ego/GSearch?query=proteolysis&mode=name) | 4 | 44.4 | 0.0034 |
| GOTERM_MF_ALL | [hydrolase activity](http://www.ebi.ac.uk/ego/GSearch?query=hydrolase activity&mode=name) | 5 | 55.6 | 0.011 |
| INTERPRO_NAME | [Peptidase, cysteine peptidase active site](http://www.ebi.ac.uk/interpro/DisplayIproEntry?ac=IPR000169) | 2 | 22.2 | 0.021 |
| SP_PIR_KEYWORDS | [hydrolase](http://us.expasy.org/cgi-bin/get-entries?KW=hydrolase) | 4 | 44.4 | 0.024 |
| UP_SEQ_FEATURE | propeptide:Activation peptide | 2 | 22.2 | 0.044 |
| GOTERM_BP_ALL | [cell-cell signaling](http://www.ebi.ac.uk/ego/GSearch?query=cell-cell signaling&mode=name) | 3 | 33.3 | 0.044 |
| GOTERM_BP_ALL | [cellular protein metabolism](http://www.ebi.ac.uk/ego/GSearch?query=cellular protein metabolism&mode=name) | 5 | 55.6 | 0.048 |
| GOTERM_BP_ALL | [cellular macromolecule metabolism](http://www.ebi.ac.uk/ego/GSearch?query=cellular macromolecule metabolism&mode=name) | 5 | 55.6 | 0.052 |
| SP_PIR_KEYWORDS | [thiol protease](http://us.expasy.org/cgi-bin/get-entries?KW=thiol protease) | 2 | 22.2 | 0.052 |
| GOTERM_MF_ALL | [cysteine-type endopeptidase activity](http://www.ebi.ac.uk/ego/GSearch?query=cysteine-type endopeptidase activity&mode=name) | 2 | 22.2 | 0.053 |
| GOTERM_MF_ALL | [cysteine-type peptidase activity](http://www.ebi.ac.uk/ego/GSearch?query=cysteine-type peptidase activity&mode=name) | 2 | 22.2 | 0.065 |
| GOTERM_BP_ALL | [protein metabolism](http://www.ebi.ac.uk/ego/GSearch?query=protein metabolism&mode=name) | 5 | 55.6 | 0.066 |
| GOTERM_MF_ALL | [catalytic activity](http://www.ebi.ac.uk/ego/GSearch?query=catalytic activity&mode=name) | 6 | 66.7 | 0.067 |
