# Supplementary material for: Estrogen, not intrinsic aging, is the major regulator of delayed human wound healing in the elderly
Source: Genome Biol. 2008 May 13;9(5):R80. doi: 10.1186/gb-2008-9-5-r80 (PMC2441466; doi:10.1186/gb-2008-9-5-r80)
Supplement: Additional data file 6 — Subset S4: GenAge-derived aging-associated probe sets. [file gb-2008-9-5-r80-S6.doc]

**Supplementary Table 6 – Subset 4 (s4): Genage-derived aging-associated probe sets that are differentially expressed in wounds from young and elderly subjects, up (green) & down (red**) in old.

| **Affy ID** | **Gene^a^** | **Gene (Description)** | **Function** | **q value^b^** | **FC^c^** |
| --- | --- | --- | --- | --- | --- |
| 210338_s_at | **HSPA8** (E) | heat shock 70kDa protein 8 | Aging-associated heat shock protein | 9.9E-04 | -10.6 |

a. Genes in **bold** have been validated by Real-time PCR.

b. CyberT-derived multiple testing corrected q-value

c. Fold change (old/young)

(E). Also estrogen-regulated (Table 1)
