# Supplementary material for: Estrogen, not intrinsic aging, is the major regulator of delayed human wound healing in the elderly
Source: Genome Biol. 2008 May 13;9(5):R80. doi: 10.1186/gb-2008-9-5-r80 (PMC2441466; doi:10.1186/gb-2008-9-5-r80)
Supplement: Additional data file 7 — Subset S5: hand-curated and aging-associated GO probe sets. [file gb-2008-9-5-r80-S7.doc]

**Supplementary Table 7 – Subset 5 (s5): Hand-curated and aging-associated GO probe sets that are differentially expressed in wounds from young and elderly subjects, up (green) & down (red**) in old.

| **Affy ID** | **Gene^a^** | **Gene (Description)** | **Function** | **q value^b^** | **FC^c^** | **GO / Ref** |
| --- | --- | --- | --- | --- | --- | --- |
| 217496_s_at | **IDE** (E) | insulin-degrading enzyme | Wound fluid / resolution of insulin response | 4.0E-06 | -20.5 | [Leissring](http://www.ncbi.nlm.nih.gov/sites/entrez?Db=pubmed&Cmd=Search&Term="Leissring MA"%5BAuthor%5D&itool=EntrezSystem2.PEntrez.Pubmed.Pubmed_ResultsPanel.Pubmed_RVAbstractPlus)., 2004 |
| 210074_at | **CTSL2** (E) | cathepsin L2 | Lysosomal cysteine proteinase | 3.8E-05 | -15.5 | Viken., 2007 |
| 214131_at | **SERPINB13** | serpin peptidase inhibitor, clad… | UV-responsive proteinase inhibitor | 1.1E-03 | -15.0 | Response to UV |
| 214131_at | C12orf5 | chromosome 12 open reading … | Protection from DNA damage | 1.1E-03 | -12.8 | Bensaad., 2006 |
| 204733_at | **KLK6** (E) | kallikrein 6 (neurosin, zyme) | Hormone regulated serine protease | 1.4E-05 | -11.9 | [Zarghooni](http://www.ncbi.nlm.nih.gov/sites/entrez?Db=PubMed&Cmd=Search&Term="Zarghooni M"%5BAuthor%5D&itool=EntrezSystem2.PEntrez.Pubmed.Pubmed_ResultsPanel.Pubmed_RVAbstractPlus)., 2002 |
| 202179_at | BLMH (E) | bleomycin hydrolase | Alzheimer’s-associated cysteine peptidase | 2.1E-03 | -11.8 | Neurodegeneration |
| 201849_at | **BNIP3** (E) | BCL2/adenovirus E1B 19kDa… | Mitochondrial apoptosis inducing protein | 2.7E-04 | -10.1 | [Rohrbach](http://www.ncbi.nlm.nih.gov/sites/entrez?Db=pubmed&Cmd=Search&Term="Rohrbach S"%5BAuthor%5D&itool=EntrezSystem2.PEntrez.Pubmed.Pubmed_ResultsPanel.Pubmed_RVAbstractPlus) et al., 2005 |
| 203328_x_at | **IDE** (E) | insulin-degrading enzyme | Wound fluid / resolution of insulin response | 1.4E-05 | -17.4 | [Leissring](http://www.ncbi.nlm.nih.gov/sites/entrez?Db=pubmed&Cmd=Search&Term="Leissring MA"%5BAuthor%5D&itool=EntrezSystem2.PEntrez.Pubmed.Pubmed_ResultsPanel.Pubmed_RVAbstractPlus)., 2004 |
| 203327_at | **ID**E (E) | insulin-degrading enzyme | Wound fluid / resolution of insulin response | 7.0E-04 | -9.3 | [Leissring](http://www.ncbi.nlm.nih.gov/sites/entrez?Db=pubmed&Cmd=Search&Term="Leissring MA"%5BAuthor%5D&itool=EntrezSystem2.PEntrez.Pubmed.Pubmed_ResultsPanel.Pubmed_RVAbstractPlus)., 2004 |
| 205016_at | TGFA (E) | transforming growth factor, alpha | IFN-induced / epidermal regeneration | 1.0E-03 | -8.5 | [Xiao](http://www.ncbi.nlm.nih.gov/sites/entrez?Db=pubmed&Cmd=Search&Term="Xiao ZQ"%5BAuthor%5D&itool=EntrezSystem2.PEntrez.Pubmed.Pubmed_ResultsPanel.Pubmed_RVAbstractPlus) et al., 2003 |
| 212907_at | **SLC30A1** | Solute carrier family 30 (zinc tr… | Zinc / Calcium ion transporter | 8.5E-04 | -7.3 | Lovell et al., 2005 |

a. Genes in **bold** have been validated by Real-time PCR.

b. CyberT-derived multiple testing corrected q-value

c. Fold change (old/young)

(E). Also estrogen-regulated (Table 1)
