# Supplementary material for: Estrogen, not intrinsic aging, is the major regulator of delayed human wound healing in the elderly
Source: Genome Biol. 2008 May 13;9(5):R80. doi: 10.1186/gb-2008-9-5-r80 (PMC2441466; doi:10.1186/gb-2008-9-5-r80)
Supplement: Additional data file 8 — Differentially expressed genes identified in this study, using a relaxed array filtering criteria, that have also been demonstrated to alter life-span in animal models. [file gb-2008-9-5-r80-S8.doc]

**Supplementary Table 8 – Identified genes that have also been demonstrated to alter life-span in animal models, up (green) & down (red**) in old.

| **Gene^a^** | **Function** | **p-value^b^** | **FC^c^** | **Animal model** | **Lifespan effect** |
| --- | --- | --- | --- | --- | --- |
| **LMNA** | Mutated in premature aging/Hutchinson-Gilford's proger. syndrome | - | **1.67** | Human | **Increase** |
| **WRN** | DNA repair enzyme. Mutated in premature aging/Werner syndrome | - | **1.63** | Human | **Increase** |
| **ERCC5/8** | Defective repair of oxidative base damage in Cockayne syndrome. | - | **1.52** | Human | **Increase** |
| HMGB1 | DNA unwinding, stress response. | 2.6E-03 | **2.73** | Indirect | **Increase** |
| NBN | Mutated in Nijmegen breakage syndrome (chromosomal instability) | 5.0E-02 | **2.52** | Indirect | **Increase** |
| JUN | JNK pathway regulates lifespan in C.elegans through FOXO3A. | 1.9E-02 | **2.36** | Indirect | **Increase** |
| SIRT2 | NAD-dependent deacetylase. | 4.8E-02 | **2.12** | C.el./Dros | **Increase** |
| H2AFX | Histone. DNA repair. | 4.5E-02 | **1.77** | Indirect | **Increase** |
| PIK3CB | age-1 homologue. Modulates plasma IGF1 levels & longevity. | 2.8E-02 | **1.69** | C.elegans | **Increase** |
| **HSPA8** | Chaperone. | 9.2E-06 | **-10.6** | Drosophila | **Increase** |
| TP73L | P63. Adhesion, apoptosis, development, wound healing, cancer | 2.1E-02 | **-4.19** | Mouse | **Increase** |
| XRCC5 | DNA repair, telomere & WRN-associated | 3.9E-04 | **-3.84** | Mouse | **Increase** |
| ATP5O | Mitochondrial. | 5.8E-03 | **-2.82** | C.elegans | **Decrease** |
| **SDHC** | Mitochondrial. Protects from oxidative stress & premature aging. | 2.6E-03 | **-2.51** | C.elegans | **Increase** |
| HSP90AA1 | Molecular chaperone. | 6.1E-03 | **-2.6** | Drosophila | **Increase** |
| HSPA1A | Chaperone | 2.4E-02 | **-2.5** | Drosophila | **Increase** |
| MAPK14 | (p38). IL6 production & cell senescence. | 2.0E-02 | **-2.44** | Senecsen. | **Decrease** |
| FOXO3A | IGF1 pathway, DAF-16 homologue with clear role in aging. | 1.6E-02 | **-2.39** | C.elegans | **Increase** |
| IRS2 | Involved in the IGF1/GH1 axis. | 8.5E-03 | **-2.35** | Drosophila | **Decrease** |
| HDAC1 | Histone deacetylase. | 1.4E-02 | **-2.14** | Drosophila | **Decrease** |
| BUB3 | Mitotic spindle checkpoint gene | 1.6E-02 | **-2.02** | Mouse | **Increase** |
| MORF4 | Cellular senescence inducing transcription factor | 3.8E-02 | **-2.0** | Senecsen. | **Decrease** |
| EEF1A1 | Protein synthesis. | 5.0E-02 | **-1.7** | Drosophila | **Increase** |

a. Genes in **bold** have been validated by Real-time PCR.

b. CyberT-derived p-value

c. Fold change (old/young)
