# Supplementary material for: Estrogen, not intrinsic aging, is the major regulator of delayed human wound healing in the elderly
Source: Genome Biol. 2008 May 13;9(5):R80. doi: 10.1186/gb-2008-9-5-r80 (PMC2441466; doi:10.1186/gb-2008-9-5-r80)
Supplement: Additional data file 9 — Primers used for qPCR. [file gb-2008-9-5-r80-S9.doc]

**Supplementary tabl**e 9 – qPCR Primers

| **Gene** | **Left Primer** | **Right Primer** |
| --- | --- | --- |
| *GPX3* | ATGCTGGCAAATACGTCCTC | AGAATGACCAGACCGAATGG |
| *LOR* | GCTCTGTCTGCGGCTACTCT | ACGAGGTCTGAGTGACCTGC |
| *ARG1* | GGAGACCACAGTTTGGCAAT | CCACTTGTGGTTGTCAGTGG |
| *SERPINB7* | AGTCAGGGCTCCAGTCTCAA | GCAAAAAGCCCATTCACAAT |
| *SLPI* | GAAATCTGCCCAGTGCCTTA | GTCAACAGGATCCAGGCATT |
| *CXCL9* | TTTTCCTCTTGGGCATCATC | GAACAGCGACCCTTTCTCAC |
| *SDHC* | GATGGAGCGGTTCTGGAATA | AGAGAGACCCCTGCACTCAA |
| *IDE* | CTTGCCACAACCTGAAGTGA | CAGAGTTTTGCAGCCATGAA |
| *WRN* | GGGGCATGATTTTAGGGATT | TTCCCGGATTGAAGAACTTG |
| *BNIP3* | ACCCTCAGCATGAGGAACAC | AGCAGCAGAGATGGAAGGAA |
| *ERCC8* | ATGTTGAAAGAATCCACGGC | CACACCATCTGAACCACCTG |
| *LMNA* | ATGGAGATGATCCCTTGCTG | AGGTGTTCTGTGCCTTCCAC |
| *DEFB4* | AAATTGGCACCTGTGGTCTC | GAGGGAGCCCTTTCTGAATC |
| *PTPRO* | CGCCTTGTCAAGCTAGAACC | CCCACAGCAAAGTCACTGAA |
| *LYPD3* | TGACTGTGTCCTTGCCTGTC | GCGGAGGTCAGAGTTACAGC |
| *CD36* | ACCATTGGTGATGAGAAGGC | GCAACAAACATCACCACACC |
| *HAL* | CTCCTGGAGCAGAAGGTTTG | TTGCAGTGAAAAGGCTGTTG |
| *DSC1* | GCAACAACTGCAGATGGCTA | TGTTCAAAATATGGGGCGTT |
| *SELE* | AGCTTCCCATGGAACACAAC | TACACTGAAGGCTCTGGGCT |
| *HSPA8* | GGAGGTGGCACTTTTGATGT | TGACCATTCGGTTGTCAAAA |
| *KLK6* | CCCTGTGGATCAAAGGAGAA | CACTTGGCCTGAATGGTTTT |
| *PEPI* | CAGGTAACAACTCCGTGGGT | ACCATAACACAGCACGTGGA |
| *COL1A1* | GTGCTAAAGGTGCCAATGGT | ACCAGGTTCACCGCTGTTAC |
| *GAPDH* | TGCACCACCAACTGCTTAGC | GGCATGGACTGTGGTCATGAG |
| *YWAHZ* | ACTTTTGGTACATTGTGGCTTCAA | CCGCCAGGACAAACCAGTAT |
| *HPRT* | TGACACTGGCAAAACAATGCA | GGTCCTTTTCACCAGCAAGCT |
| *18S* | AGTCCCTACCCTTTGTACACA | GATCCAAGGGCCTCACTAAAC |
| *Ltf (mouse)* | TGACAAACAGAGCTGATGCC | AGACTTCAGCTGCCACAGGT |
| *Defb4 (mouse)* | ACCAGGCTTCAGTCATGAGG | TTTGGGTAAAGGCTGCAAGT |
| *Arg1 (mouse)* | AAAGCTGGTCTGCTGGAAAA | ACAGACCGTGGGTTCTTCAC |
| *Ptpro (mouse)* | GTTGCAGAACTGAAGGAGCC | GCTTTTCCGAGTCTCACAGG |
| *Hop (mouse)* | AACAAGGTCAACAAGCACCC | AAACCATTTCTGCGTCTGCT |
| *Lypd3 (mouse)* | TCCTGGCCTTCTTTCAGCTA | TATGCACTCTCATTGCCTGC |
| *Cd36 (mouse)* | TGGGAAGACAATCAAAAGGG | TGGGTTTTGCACATCAAAGA |
| *Sprr1a (mouse)* | CCCCTCAACTGTCACTCCAT | CAGGAGCCCTTGAAGATGAG |
| *Hal (mouse)* | CGTTCCCATTCTTCAGGTGT | TCTCCAAGGAAATCCCACTG |
| *Serpinb3 (mouse)* | ATTTTGCACATGCAGCTGAG | GCTACTGTTCAGGCTCCCAC |
| *Serpinb7 (mouse)* | TTTAGGTCTCCCACGTGTCC | ACATGCTTATGCCACCATGA |
| *Dsc1 (mouse)* | AGAAGAAGTGACGGAAGCCA | ACCACCAAGAGTCCCAACAG |
| *Cxcl9 (mouse)* | CAAAATTTCATCACGCCCTT | CCAGACAGCTGTTGTGCATT |
| *Sele (mouse)* | AGCTACCCATGGAACACGAC | CGTTATCCCAGATGCCAGAT |
| *Bnip3 (mouse)* | AATACTGTTGGATGCCCAGC | ATCTTGTGGTGTCTGGGAGC |
| *Hspa8 (mouse)* | GGAGGTGGCACTTTTGATGT | TGACCATTCGGTTGTCAAAA |
| *Ide (mouse)* | GTGAACGCTGTCGATTCAGA | ATTTGCTGAAGGGGTGTTTG |
| *Klk6 (mouse)* | TGTGCTGATGTCCATCTGGT | AATCACCCTGACAGGAATCG |
| *Blmh (mouse)* | ACAACCAGCCCATTGACTTC | AACATCACAGCCAAACCACA |
| *Gapdh (mouse)* | CCCACTAACATCAAATGGGG | TCTCCATGGTGGTGAAGACA |
| *Ywahz (mouse)* | TTCTTGATCCCCAATGCTTC | TTCTTGTCATCACCAGCAGC |
| *Hprt (mouse)* | TGCTCGAGATGTCATGAAGG | AATCCAGCAGGTCAGCAAAG |
